# Chatbot-Delivered Stage of Change–Tailored Web-Based Intervention to Promote Physical Activity Among Inactive Community-Dwelling People Aged 65 years or More: Protocol for a Randomized Controlled Trial

**DOI:** 10.2196/68796

**Published:** 2025-06-20

**Authors:** Xue Liang, Fenghua Sun, Qingpeng Zhang, Yuan Fang, Fuk-yuen Yu, Danhua Ye, Borui Zhang, Qianwen Liao, Phoenix KH Mo, Zixin Wang

**Affiliations:** 1 Centre for Health Behaviours Research Jockey Club School of Public Health and Primary Care Chinese University of Hong Kong Hong Kong China (Hong Kong); 2 Department of Health and Physical Education Education University of Hong Kong Tai Po China (Hong Kong); 3 Musketeers Foundation Institute of Data Science University of Hong Kong Hong Kong China (Hong Kong); 4 Department of Pharmacology and Pharmacy LKS Faculty of Medicine University of Hong Kong Hong Kong China (Hong Kong)

**Keywords:** chatbot, physical activity, intervention, randomized controlled trial, older adult, stage of change, motivation

## Abstract

**Background:**

Physical activity (PA) has significant health benefits for older adults. However, many older adults in Hong Kong remain physically inactive. Interventions tailored to one’s current stage of change (SOC) are more effective than non-SOC-tailored ones in facilitating behavioral changes. Chatbots are potentially useful to deliver SOC-tailored interventions to promote PA among older adults.

**Objective:**

This randomized controlled trial (RCT) will compare the efficacy of an SOC- versus a non-SOC-tailored intervention in increasing the prevalence of meeting World Health Organization (WHO)-recommended PA levels 6 months after completion of the intervention among inactive community-dwelling individuals aged ≥65 years.

**Methods:**

This is a partially blinded (outcome assessors and data analysts) and parallel-group RCT. A total of 278 inactive community-dwelling people aged 65 years or more will be randomized evenly into either an intervention group or a control group. In the intervention group, a fully automated chatbot with natural language processing (NLP) functions will measure participants’ SOC related to PA and deliver web-based interventions tailored to their current SOC every week for 12 weeks. In the control group, the chatbot will not measure participants’ SOC but will deliver a non-SOC-tailored web-based intervention every week for 12 weeks. Participants will be interviewed at baseline (T0), after completion of the intervention (T1), and 6 months after T1 (T2). The primary outcome is the prevalence of meeting WHO-recommended PA levels (ie, at least 150 minutes of moderate-intensity aerobic PA, at least 75 minutes of vigorous-intensity aerobic PA, or an equivalent combination of moderate-to-vigorous physical activity [MVPA] every week). PA will be measured using the Chinese version of the International Physical Activity Questionnaire Short Form (IPAQ-SF) and accelerometers at T0, T1, and T2. Secondary outcomes include (1) minutes of MPVA, low-intensity PA, and sedentary time in the past week; (2) step counts in the past week; (3) SOC levels, perceived pros, perceived cons, and perceived self-efficacy related to PA; (4) compliance to the web-based interventions; and (5) cognitive status measured at T0, T1, and T2. Intention-to-treat analysis will be used for data analysis.

**Results:**

Recruitment started in November 2024. By February 2025, a total of 185 participants completed the baseline assessment and were randomly assigned to either the intervention group (n=93, 50.3%) or the control group (n=92, 49.7%). Recruitment will be completed by the end of June 2025. The follow-up assessment at T1 started in March 2025. Data collection is expected to be concluded in February 2026.

**Conclusions:**

The findings will extend the application of SOC and contribute to the evidence of the effectiveness of SOC-tailored and chatbot-delivered interventions. If the chatbot-delivered SOC-tailored intervention is proven effective to increase PA levels, it will require relative less resources to implement and maintain. It can be integrated into the existing WhatsApp groups operated by organizations providing services to older adults in Hong Kong and create public health impacts.

**Trial Registration:**

ClinicalTrial.gov: NCT06641492; https://clinicaltrials.gov/study/NCT06641492

**International Registered Report Identifier (IRRID):**

DERR1-10.2196/68796

## Introduction

### Background

Hong Kong has a rapidly aging population. It is predicted that 22% of Hong Kong residents will be 65 years or older by 2030 [1]. Recent data show that 75% of Hong Kong older adults have one or more chronic diseases [1], which has created a huge burden on the health care system.

Physical activity (PA) is defined as any bodily movement produced by skeletal muscles, resulting in an expenditure of energy [2]. PA is well recognized as an effective intervention for reducing mortality and dependence-inducing diseases [3] and improving cognitive function, frailty symptoms, and physical functions among older adults [4,5]. The World Health Organization (WHO) recommends that older adults without any contradiction of PA do at least 150 minutes of moderate-intensity aerobic PA, 75 minutes of vigorous-intensity aerobic PA, or an equivalent combination of moderate-to-vigorous physical activity (MVPA) every week [3]. However, physical inactivity remains a worldwide phenomenon and increases significantly with age. A systematic review showed that 43.4%-78% of older adults across countries cannot meet the WHO-recommended PA levels [6]. In Hong Kong, the proportion of older adults who did not meet the WHO-recommended PA level increased from 13.5%-42.8% in 2018-2019 [7] to 22.2%-53.3% in 2020-2022 [8]. A recently published study showed that 28.6% of older adults in Hong Kong had a low level of PA in July 2022 [9]. There is a strong need for improvement.

Factors associated with PA among older adults have been well studied. A systematic review suggested that a lack of knowledge, skills, capacities, or support from peers or family members related to PA, the perception that PA would cause pain or injury, difficulties in accessing sports facilities, and concerns related to bad weather are barriers to performing PA among older adults [10]. Perceived benefits of PA on physical and mental health, perceived self-efficacy to perform PA, and suggestions from health professionals have been identified as facilitators of PA in this population [10]. Similar facilitators and barriers apply to older adults in Hong Kong [9]. These factors will be addressed by our interventions.

A number of interventions have been conducted to promote PA among older adults. The majority of them use face-to-face approaches, such as motivational interviewing, PA coaching, and educational sessions [11]. As compared to no or minimum interventions, these approaches increase objectively measured PA levels in both the short term (≤3 months) and the medium term (3-12 months) [11]. However, these interventions require significant personnel and resources to implement. There is an increasing number of web-based interventions for older adults. A systematic review of 18 randomized controlled trials (RCTs) showed that web-based interventions are feasible to promote PA among older adults [2]. Digital PA tracking and feedback (ie, providing automatic tracking and feedback on PA levels based on pedometers/accelerometers/smartphones) and digital PA coaching (ie, goal setting, promoting PA according to participants’ baseline PA situation) were the most commonly used strategies in these RCTs [2]. However, 7 of these RCTs were feasibility trials with limited sample sizes (10-30 per group) instead of full-powered RCTs, and 11 only measured short-term efficacy (≤3 months). Moreover, none of these RCTs was conducted with the Chinese population.

As the most commonly used stage model, the stage of change (SOC) postulates that completed behavioral changes progress through 5 ordinal stages (precontemplation stage, contemplation stage, preparation stage, action stage, and maintenance stage), and individuals require interventions tailored to their current SOC in order to effectively facilitate progression toward sustained behavioral changes [12]. A meta-analysis showed that SOC-tailored interventions are more effective than non-SOC-tailored ones [13]. The SOC has been widely applied to guide PA promotion in different populations [14-23], including older adults [19,20,22,23]. However, most of the SOC-tailored PA interventions use face-to-face approaches (eg, counselling, written material, onsite educational sessions, and motivational interviews) [14-16,18,19,21,23]. It is resource demanding as project staff need to measure the SOC of each participant and prepare and select interventions tailored to their current SOC. To the best of our knowledge, only 2 web-based PA interventions targeting older adults have tried to send some simple tips in text messages tailored to their current SOC [20,22].

Chatbots are computerized programs that replicate human interactions through various forms of communication [24], and they have become increasingly popular in health communication. Systematic reviews have highlighted the feasibility and effectiveness of chatbots in promoting healthy lifestyles, smoking cessation, treatment adherence, and vaccination uptake and reducing substance misuse [25-27]. Two studies applied chatbots to promote PA among office workers in South Korea [28] and adults in the United States [29]. In the South Korean study, the chatbot automatically sent a message to each participant every day to remind them to perform the stair-climbing activity. Once the participant completed the desired activity and notified the chatbot, the chatbot automatically provided positive feedback to the participant. In addition, coffee coupons were given to those who completed this activity. This intervention was shown to be effective in increasing participants’ stair-climbing behaviors [28]. The other study conducted in the United States used a chatbot to provide knowledge and counselling related to PA, which was effective in increasing participants’ daily step count [29]. Chatbots can proactively interact with users to determine their SOC and automatically select a pathway of intervention that is tailored to each user’s current SOC, thus making chatbots ideal for delivering SOC-tailored interventions to promote PA. A previous study demonstrated that applying chatbots to deliver health promotion is feasible and acceptable among older adults in Hong Kong [30].

### Objectives

The proposed RCT will compare the efficacy of an SOC- versus a non-SOC-tailored intervention in increasing the prevalence of meeting WHO-recommended PA levels 6 months after completion of the intervention among inactive community-dwelling individuals aged ≥65 years. Given the strengths of chatbots in health promotion, both the SOC- and non-SOC-tailored interventions will be delivered by a chatbot with natural language processing (NLP) functions. The PA levels will be measured using the validated Chinese version of the International Physical Activity Questionnaire Short Form (IPAQ-SF) and accelerometers.

The secondary objectives of this RCT are to compare the following outcomes 6 months after completion of the intervention between the intervention and control groups: (1) minutes of MPVA, low-intensity PA, and sedentary time in the past week measured using both the IPAQ-SF and accelerometers; (2) step counts in the past week measured using accelerometers; (3) SOC levels, perceived pros, perceived cons, and perceived self-efficacy related to PA; (4) compliance to the web-based interventions; and (5) cognitive status.

## Methods

### Study Design

**Figure 1:**
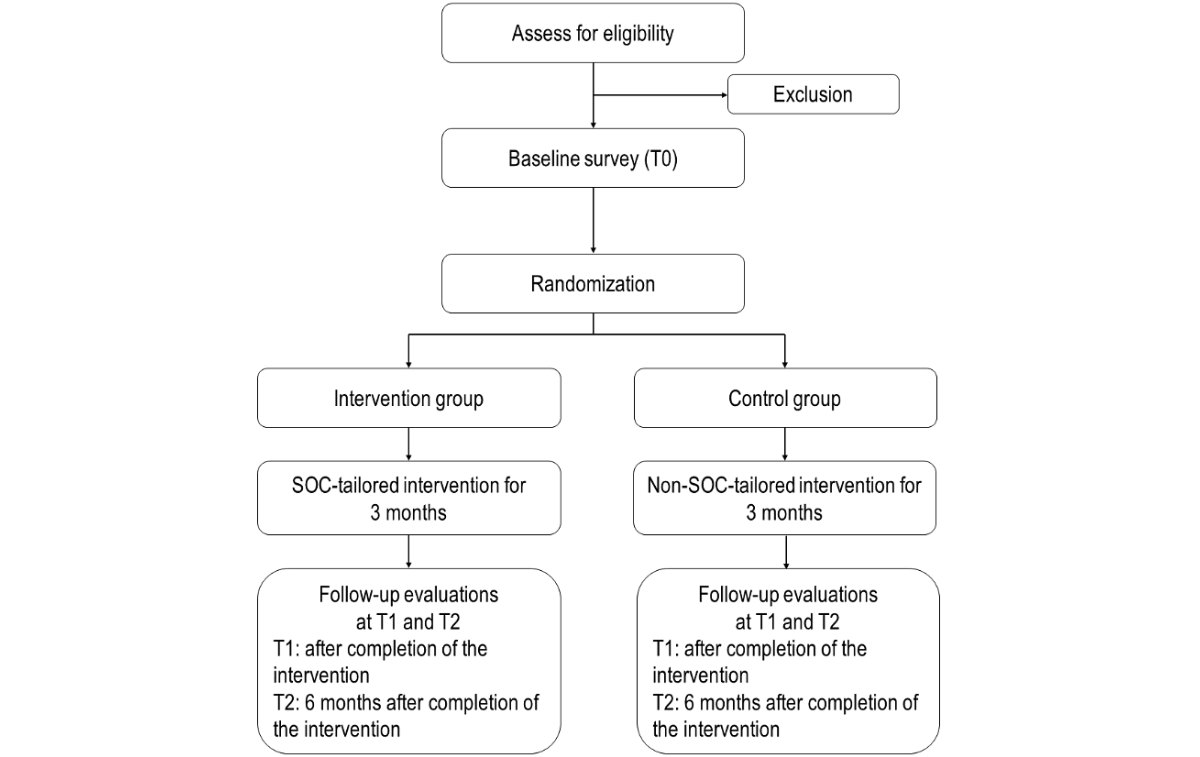
Flowchart of the RCT. RCT: randomized controlled trial; SOC: stage of change.

This is a partially blinded (outcome assessors and data analysts) and parallel-group RCT to be conducted in Hong Kong, China. Inactive community-dwelling people aged 65 years or more will be randomized evenly (1:1) into either an intervention group or a control group. In the intervention group, the chatbot will measure participants’ SOC related to PA and deliver web-based interventions tailored to their current SOC every week for 12 weeks. In the control group, the chatbot will not measure participants’ SOC but will deliver a standard and non-SOC-tailored web-based intervention every week for 12 weeks. Participants will be interviewed at baseline (T0), after completion of the intervention (T1), and 6 months after T1 (T2). The study was registered at ClinicalTrials (NCT06641492). The study flowchart diagram is shown in Figure 1. We will report this trial in accordance with the CONSORT-EHEALTH (Consolidated Standards of Reporting Trials of Electronic and Mobile Health Applications and Online Telehealth) checklist (Multimedia Appendix 1) [31] and the SPIRIT (Standard Protocol Items: Recommendations for Interventional Trials) checklist (Multimedia Appendix 2).

### Participants and Recruitment

Inclusion criteria are as follows:

- Age 65 years or more.
- Having a Hong Kong identity card.
- Chinese speaking.
- Not meeting the WHO-recommended level of MVPA, defined as <150 minutes of moderate-intensity aerobic PA in the past week. The interviewers will assess participants using the validated Chinese version of the IPAQ-SF.
- Willing to be followed over the phone at T1 and T2.
- Owning a smartphone.
- Able to send and read text/voice messages via smartphones.
- Willing to wear an accelerometer for a week at T0, T1, and T2.

Exclusion criteria include the following:

- Blindness or deafness
- Diagnosis of major psychiatric diseases (schizophrenia or bipolar disorders) or dementia
- Score≥1 in the Chinese version of the Physical Activity Readiness Questionnaire (PAR-Q)
- Score≤16 in the validated telephone version of the Cantonese Mini-mental State Examination (T-CMMSE) [32]

Participants will be recruited through random telephone calls. Telephone numbers will be selected from up-to-date Hong Kong telephone directories. Trained interviewers will call potential participants over the phone. If there is more than one person in the household who is aged ≥65 years, the one whose birthday is closest to the interview date will be invited for the study to avoid contamination and introduction of extra confounding factors. Interviewers will brief prospective participants about the study and screen their eligibility.

### Sample Size Planning

At T0, no participant will meet the WHO-recommended PA level according to the inclusion criteria. We assume that 10% of participants in the control group will meet the WHO-recommended PA level at T2 as there are minimum interventions. A target sample size of 97 per group (194 in total) would allow us to detect a between-group difference of 15% in the prevalence of meeting the WHO-recommended PA level at T2 (25% versus 10%; power=0.80, α=.05, PASS version 11.0 [NCSS Statistical Software]). We conservatively estimate the dropout rate to be 30% at T2. Thus, 139 eligible participants per group (278 in total) are required.

### Ethical Considerations

Ethics approval was obtained from the Survey and Behavioural Research Ethics Committee of the Chinese University of Hong Kong (approval number SBRE-22-0257) and the Joint Chinese University of Hong Kong – New Territories East Cluster Clinical Research Ethics Committee (approval number 2022.610-T). All participants will fill out an electronic consent form through a link sent over WhatsApp to confirm their informed consent. The electronic consent forms and participants’ contact information will be kept separately from the questionnaires in a locked cabinet in the office of the principal investigator. Participants’ names and contact information will not appear on the questionnaire or in the dataset. A unique study ID will be assigned to each participant throughout the study. The dataset will be protected by a password on the computer of the principal investigator. All data will be kept for 5 years after conclusion of the project. The data will be disposed of or deleted securely and confidentially once the retention period has expired.

A supermarket coupon worth HK $50 (US $6.37) will be mailed to an address provided by the participants upon completion of the surveys at T0, T1, and T2. A supermarket coupon worth HK $150 (US $19.11) will be given to the participants in the same manner after they finish wearing and return the accelerometers at T0, T1, and T2.

### Development and Maintenance of the Chatbot

**Figure 2:**
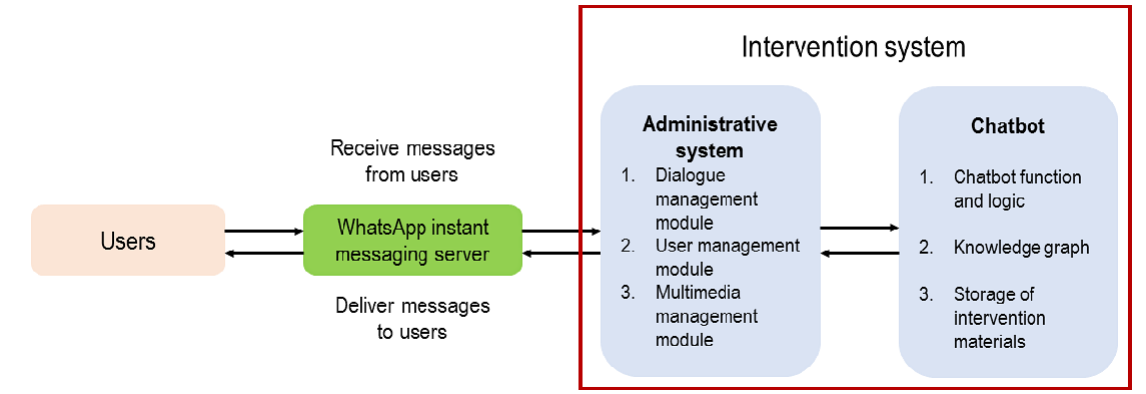
Architecture of the chatbot system.

A chatbot will be developed and maintained on the WhatsApp platform due to its wide popularity among local older adults and a user-friendly interface. The architecture of the chatbot system is shown in Figure 2. Users send information to the WhatsApp instant messaging server, which then delivers the message to the chatbot. The chatbot system processes and sends a message back to WhatsApp, where users can view the information. The knowledge graph, which links participants’ input with the chatbot’s responses, will be prepared by focus groups of 30 older adults and interviews with experts in sports medicine. The knowledge graph will evolve over time with an increasing number of human-chatbot interactions. The chatbot will use a mixture of techniques so that the human-chatbot interactions are not merely rule based, and the chatbot will also learn from the evolving knowledge graph through machine learning. We will pilot the chatbot among another 30 older adults to improve its accuracy of human-chatbot interactions. These measures will allow the chatbot to have adequate accuracy and consistency in delivering interventions according to the protocol and prevent the chatbot becoming repetitive and off-topic after extensive usage. The same professional team will maintain the health status of the chatbot during the intervention period. A monthly review of the chatbot’s performance will be conducted.

### Baseline Survey and Randomization

Participants will be interviewed over the phone to record their background characteristics and potential confounders at baseline. Their PA in the first week after the baseline survey will be measured using an accelerometer. At the end of the baseline survey, the interviewers will help participants install WhatsApp on their smartphones, if needed. Computer-generated random allocation codes will be produced and sealed in opaque envelopes by a research staff member, without involvement in recruitment or the baseline survey. One envelope will be drawn and opened by the interviewer, who will then inform each participant about their assigned group. Block randomization with a block size of 8 will be used. Our project staff will connect participants’ WhatsApp with the chatbot, and the chatbot will automatically run a training module guiding users in an older adult–friendly way when participants access it for the first time.

### The Intervention Group

The interventions will start after participants complete baseline PA measurements with the accelerometer. The chatbot will deliver interventions cocreated by researchers and older adults based on the behavioral change strategies recommended by the SOC-tailored intervention to address the determinants of PA among older adults identified by previous studies. Twelve weekly sessions of the SOC-tailored intervention will be delivered by the chatbot within 3 months through WhatsApp. In previous studies, 3 months was the most commonly selected intervention duration for web-based interventions promoting PA among older adults [2]. Moreover, 1 study suggested that some older adults would lose interest if the duration of web-based interventions was longer than 3 months [20]. Multimedia Appendix 3 shows the workflow of the intervention group. The intervention steps are described next:

- Step 1: Initiate the intervention. The chatbot will greet participants and state its function and topics to be covered.
- Step 2: Measure the PA level. The chatbot will ask participants to fill out the Chinese version of the IPAQ-SF. The chatbot will automatically calculate their MVPA level and provide feedback based on that level.
- Step 3: Identify the SOC. The chatbot will ask participants a validated multiple-choice question [20] that has been widely used to identify the SOC related to PA: Which sentence best describes your current PA situation? The response categories are “I participate in PA for less than 150 minutes per week and do not intend to increase that time in the next 6 months” (precontemplation stage), “I participate in PA for less than 150 minutes per week and intend to increase that time in the next 6 months” (contemplation stage), “I participate in PA for less than 150 minutes per week and intend to increase that time in 1 month” (preparation stage), ”I participate in PA for at least 150 minutes per week, but I have only begun to do so in the past 6 months” (action stage), and “I participate in PA for at least 150 minutes per week and have been doing so for more than 6 months” (maintenance stage).
- Step 4: Deliver interventions tailored to participants’ current SOC levels. The intervention will address known determinants of PA among older adults identified in a systematic review and a local study [9,10]. Barriers to PA among older adults include a lack of knowledge, skills, capacities, or support from peers or family members related to PA, the perception that PA would cause pain or injury, difficulties in accessing sports facilities, and concerns about bad weather. The facilitators include perceived benefits of PA on physical and mental health, perceived self-efficacy to perform PA, and suggestions from health professionals.

#### The Precontemplation Stage

Participants in the precontemplation stage will watch a web-based video in which a sport scientist explains the definition of PA, emphasizes adequate levels of MVPA important for physical and mental health, and introduces levels of MVPA recommended by WHO for older adults. This content seeks to increase participants’ awareness of the importance of adequate MVPA.

#### The Contemplation Stage

Participants in the contemplation stage will watch a different web-based video. The same sports scientist will talk about the benefits of adequate MVPA for older adults (eg, reducing dependence-inducing diseases, such as cardiovascular diseases and cancers, and improving cognitive function, frailty syndrome, performance of physical functions, and mental health), address physical and environmental barriers to PA by introducing feasible options of PA suitable for older adults that can be performed at home or in the community, and give recommendations and encourage older adults to make a plan for PA. In addition, several older adults who perform MVPA regularly will share their positive experiences during PA (eg, better physical functions, a stronger immune system, and a positive psychological status) and how they overcame physical and environmental barriers of PA. This content aims to increase self-efficacy and perceived pros and reduce perceived costs of PA.

#### The Preparation Stage

The chatbot will provide a dropdown list of easy and enjoyable PA options that are suitable for older adults, which will be identified by focus groups of older adults and suggestions of the sports scientist, in the preparation stage. These options will include brisk walking, aerobics, stretching, strength/muscle training, Tai Chi, and 8-section brocade exercise. Participants will be invited to select one or more options from the list. The chatbot will retrieve a predesigned demonstration video related to each option and send it to participants. This content seeks to assist participants in developing and implementing an action plan, as well as increase their perceived self-efficacy related to PA.

#### The Action or Maintenance Stage

The chatbot will provide feedback and reinforcement based on participants’ PA level in the previous week. Positive reinforcement will be provided to participants who are on track to obtain or have already achieved the WHO-recommended MVPA level. This content aims to facilitate the maintenance of MVPA and to avoid relapse.

To prevent participants from watching the same video or reading the same information twice, slightly different videos/information corresponding to each SOC will be prepared. In each session, the chatbot will randomly select one for the participants. Participants in all stages can select one or more problems about PA from a list of common problems identified through a literature review and a focus group of older adults. Participants can also raise problems that are not on the list, verbally or in written form. The chatbot will retrieve relevant information from the knowledge graph and prepare a response.

### The Control Group

First, the chatbot will ask participants to fill out the Chinese version of the IPAQ-SF, automatically calculate their MVPA, and provide feedback based on the PA level. Second, the chatbot will automatically send a short web-based video covering general information of PA for older adults (eg, recommended MVPA level, suitable PA options) every week from week 1 to week 12. Finally, participants will select problems about PA from the same list in the intervention group. They will also have the option to verbally or in written form communicate any issues not included on the list. The chatbot will extract pertinent information from the same knowledge graph to craft a tailored response. Multimedia Appendix 4 shows the workflow of the control group.

If participants in the intervention or the control group provide no response to the intervention initiated by the chatbot within 24 hours, the chatbot will automatically send out 4 separate reminders. The project staff will proactively contact participants who provide no response to any reminders through WhatsApp or over the phone to identify reasons and solve problems.

### Measurements

#### Primary Outcome

Prevalence of meeting WHO-recommended levels of PA (at least 150 minutes of moderate-intensity aerobic PA, at least 75 minutes of vigorous-intensity aerobic PA, or an equivalent combination of MVPA every week) is the primary outcome. PA will be measured using the data provided by the participants in the Chinese version of the IPAQ-SF and from the accelerometers (ActiGraph) at T0, T1, and T2. After completion of the baseline telephone survey and before the randomization takes place, participants will be invited to visit our research office to collect the accelerometers. Onsite, participants will be instructed to wear the accelerometers on their waist (ie, above the right hipbone) with an elastic belt for 7 days, including 5 weekdays and 2 weekends, to collect objective data of PA levels. Participants will be asked to wear the accelerometers at all times except when they are showering or swimming. In addition, participants will be reminded to maintain their daily routine when they are wearing the accelerometers. Participants will visit our research office again 1 week afterward to return the accelerometers and recall their PA levels in the past week by completing the Chinese version of the IPAQ-SF. Similar procedures for collecting and returning accelerometers and completing the IPAQ-SF will be arranged after completion of the telephone survey at T1 and T2. The time on and time off related to accelerometer wearing each day will be recorded, and the data will be used to estimate the time spent in sedentary behavior, light PA, and MVPA. To ensure the accuracy of data that represent the habitual PA levels of participants, data from participants whose accelerometer-wearing time is less than 600 minutes per day will be considered invalid. We will calculate the sedentary time, light PA time, and MVPA time using triaxial cut points. The sedentary time, light PA time, and MVPA time have been defined as <200, <1951, and >1952 counts/minute, respectively, with the epoch length being 60 seconds [33]. The chatbot will automatically record the time spent by each participant with it.

#### Secondary Outcomes

The following secondary outcomes will be measured at T0, T1, and T2:

- Step counts and minutes of MVPA, light PA, and sedentary time in the past week measured using accelerometers.
- Compliance with the web-based interventions, including the number of intervention sessions started and completed, as documented by the chatbot.
- The SOC related to the PA measured using a validated multiple-choice question [34]. The measurement and definition of the SOC will be the same as those used by the chatbot. In addition, we will extract the SOC measured by the chatbot at the beginning of each intervention session in the intervention group from the chat history.
- Perceived pros and cons related to PA will be measured using the validated questionnaire on decisional balance developed by Nigg et al [35], which consists of 10 questions with 2 scales (pros and cons).
- Perceived self-efficacy of PA will be measured using a validated 6-item scale developed by Marcus et al [34].
- Cognitive status will be measured using cognitive tasks, including the sustained attention test [36], working memory [37], inhibitory control [38], and cognitive flexibility [39].

The outcome assessors and data analysts will be blinded to the treatment allocation. Participants will be reminded not to disclose the intervention they receive to assessors.

### Safety Issues and Adverse Events

Participants can report adverse events related to PA through WhatsApp or a hotline during the study period. If sustained serious adverse events are found, advice will be sought from a sport science expert.

### Pilot Study

A pilot study randomized 6 eligible participants into 2 groups and tested the logistics of the interventions. Feedback was collected from the participants. The results showed that the chatbot operates smoothly, and participants expressed satisfaction with its performance and interface.

### Statistical Analysis

Intention-to-treat analysis will be performed. The Markov chain Monte Carlo method will be used to impute missing primary and secondary outcomes. Variables to impute the missing values include participants’ background characteristics and baseline values of these outcomes. Chi-square and independent sample *t* tests will be performed to examine the between-group balance of potential confounders at baseline. We will compare between-group differences in primary and secondary outcomes at both T1 and T2. The relative risk, absolute risk reduction, and the number needed to be treated and their 95% CI values will be calculated using Microsoft Excel. Logistic regression for binary variables and linear regression models for continuous variables will be used to test between-group differences in primary and secondary outcomes after controlling for any baseline confounders, with *P*<.20 in between-group comparisons. IBM SPSS version 26.0 and R version 4.3.1 (R Foundation for Statistical Computing) will be used for data analysis, with 2-sided *P*<0.05 considered statistically significant.

## Results

Study recruitment started in November 2024. By the end of February 2025, a total of 185 participants completed the baseline assessment and were randomly assigned to either the intervention group (n=93, 50.3%) or the control group (n=92, 49.7%). Recruitment will be completed by the end of June 2025. The follow-up assessment at T1 started in March 2025. Data collection is expected to be concluded in February 2026.

## Discussion

### Summary

We anticipated that a higher proportion of participants in the intervention group would meet the WHO-recommended level of PA at T2 compared to the control group. The underlying purpose of this study is to prevent dependence-inducing diseases (eg, cardiovascular diseases, cancer) and improve cognitive function, frailty symptoms, body composition, and the performance of various physical functions by increasing PA levels among individuals aged 65 years or more in Hong Kong. About half of the older adults in Hong Kong did not meet the WHO-recommended PA level during 2020-2022 [8].

The proposed intervention has strong applications in healthy aging and noncommunicable disease control in Hong Kong. PA is an effective intervention to prevent dependence-inducing diseases and improve cognitive function, frailty symptoms, body composition, and the performance of various physical functions. Effective and sustainable interventions promoting PA are greatly needed but are lacking. We will develop an SOC-tailored web-based intervention and compare its efficacy with a non-SOC-tailored web-based intervention by performing an RCT, which is the most rigorous evaluation method and provides the highest quality of evidence. Considering participants may move forward to a later SOC, go backward to an earlier SOC, or stay in the same SOC after being exposed to an intervention, our intervention will have 12 sessions, and each session will be tailoring to the participants’ current SOC. All interventions will be delivered by a chatbot that integrates with WhatsApp. It can identify users’ SOC regarding PA and automatically deliver interventions corresponding to their SOC. Moreover, with evolving knowledge graphs and machine learning, the chatbot can ensure accuracy of human-machine interactions, which further increases its efficacy. Making use of a chatbot can also increase compliance to web-based interventions. Since the maintenance cost of the chatbot is relatively low, it is especially suitable to deliver our proposed intervention. The sustainability of our intervention is expected to be high. The intervention, if found to be significant and translated into service, would reduce the burden on the health care system and the cost in terms of medication, rehabilitation, and institutional care for chronic diseases.

### Limitations

This study has some limitations. First, we do not have a control group that does not use the chatbot. This RCT cannot provide evidence to support whether the intervention delivered by the chatbot will be more effective than the intervention delivered without a chatbot. Second, the intervention is limited to people aged 65 years or more who have access to smartphones. In Hong Kong, 73% of adults in this age group had smartphones in 2021 [40]. Smartphone ownership has been increasing sharply. Third, participants and those who refuse to participate in this study may have different characteristics and motivation to perform PA. We are not able to collect information from refusals, and selection bias exists. Moreover, compared to other RCTs promoting PA among older adults [2], our follow-up period is shorter (6 months versus 12 months). We have selected a shorter follow-up period due to some practical concerns. The effect size of the intervention would be smaller, and the dropout rate would be higher if we choose a longer follow-up period. Therefore, we need a much larger sample size, which is not feasible under the current funding scope. The limited ability to observe long-term effects of the interventions due to the relatively short follow-up period is a limitation of this study. Moreover, it is possible that participants’ behaviors would be altered while wearing accelerometers. A meta-analysis showed that when an accelerometer provides real-time monitoring and feedback of PA levels, using it alone or together with other interventions would result in improvement in PA among older adults [41]. However, the accelerometer will only be used to record participants’ PA levels in this study. It will not provide any feedback, and the participants will not be able to access accelerometer data during the assessment period. A previous study showed that when an accelerometer is only used for outcome assessment, there is no difference in PA levels between those who wear accelerometers and those who do not [42]. In previous RCTs, an accelerometer has been commonly used to measure older adults’ PA levels [43].

### Conclusion

In conclusion, this study will generate theoretical and practical implications. The findings will extend the application of the SOC and contribute to the evidence of the effectiveness of SOC-tailored and chatbot-delivered interventions. If the chatbot-delivered SOC-tailored intervention is proven effective to increase PA levels, it will require relative less resources to implement and maintain. It can be integrated into existing WhatsApp groups operated by organizations providing services to older adults in Hong Kong and create public health impacts.

## Appendix

Multimedia Appendix 1 CONSORT-eHEALTH checklist (V 1.6.1).

Multimedia Appendix 2 SPIRIT (Standard Protocol Items: Recommendations for Interventional Trials) checklist.

Multimedia Appendix 3 Workflow of the intervention group.

Multimedia Appendix 4 Workflow of the control group.

Multimedia Appendix 5 Peer-review report from the Health and Medical Research Fund, Health Bureau, Research Council, Hong Kong Special Administrative Region.

## Abbreviations

IPAQ-SF: International Physical Activity Questionnaire Short Form
MVPA: moderate-to-vigorous physical activity
NLP: natural language processing
PA: physical activity
RCT: randomized controlled trial
SOC: stage of change
WHO: World Health Organization

## Data Availability

The datasets to be generated or analyzed during this study will be available from the corresponding author upon reasonable request.
