# Supplementary material for: Chatbot-Delivered Stage of Change–Tailored Web-Based Intervention to Promote Physical Activity Among Inactive Community-Dwelling People Aged 65 years or More: Protocol for a Randomized Controlled Trial
Source: JMIR Res Protoc. 2025 Jun 20;14:e68796. doi: 10.2196/68796 (PMC12228010; doi:10.2196/68796)
Supplement: Multimedia Appendix 1 [file resprot_v14i1e68796_app1.pdf]

CONSORT-EHEALTH (V 1.6.1) - Submission/Publication Form

The CONSORT-EHEALTH checklist is intended for authors of randomized trials evaluating web-based and Internet-based applications/interventions, including mobile interventions, electronic games (incl multiplayer games), social media, certain telehealth applications, and other interactive and/or networked electronic applications. Some of the items (e.g. all subitems under item 5 - description of the intervention) may also be applicable for other study designs.

The goal of the CONSORT EHEALTH checklist and guideline is to be
a) a guide for reporting for authors of RCTs,
b) to form a basis for appraisal of an ehealth trial (in terms of validity)

CONSORT-EHEALTH items/subitems are MANDATORY reporting items for studies published in the Journal of Medical Internet Research and other journals / scientific societies endorsing the checklist.

Items numbered 1., 2., 3., 4a., 4b etc are original CONSORT or CONSORT-NPT (non-pharmacologic treatment) items.
Items with Roman numerals (i., ii, iii, iv etc.) are CONSORT-EHEALTH extensions/clarifications.

As the CONSORT-EHEALTH checklist is still considered in a formative stage, we would ask that you also RATE ON A SCALE OF 1-5 how important/useful you feel each item is FOR THE PURPOSE OF THE CHECKLIST and reporting guideline (optional).

Mandatory reporting items are marked with a red *.
In the textboxes, either copy & paste the relevant sections from your manuscript into this form - please include any quotes from your manuscript in QUOTATION MARKS,
or answer directly by providing additional information not in the manuscript, or elaborating on why the item was not relevant for this study.

YOUR ANSWERS WILL BE PUBLISHED AS A SUPPLEMENTARY FILE TO YOUR PUBLICATION IN JMIR AND ARE CONSIDERED PART OF YOUR PUBLICATION (IF ACCEPTED).
Please fill in these questions diligently. Information will not be copyedited, so please use proper spelling and grammar, use correct capitalization, and avoid abbreviations.

DO NOT FORGET TO SAVE AS PDF _AND_ CLICK THE SUBMIT BUTTON SO YOUR ANSWERS ARE IN OUR DATABASE !!!

Citation Suggestion (if you append the pdf as Appendix we suggest to cite this paper in the caption):
Eysenbach G, CONSORT-EHEALTH Group
CONSORT-EHEALTH: Improving and Standardizing Evaluation Reports of Web-based and Mobile Health Interventions
J Med Internet Res 2011;13(4):e126

URL: http://www.jmir.org/2011/4/e126/
doi: 10.2196/jmir.1923
PMID: 22209829

wangzx@cuhk.edu.hk Switch account

Not shared

Draft saved

* Indicates required question

Your name *

First Last

Zixin Wang

Primary Affiliation (short), City, Country *

University of Toronto, Toronto, Canada

The Chinese University of Hong Kong

Your e-mail address *

abc@gmail.com

wangzx@cuhk.edu.hk

Title of your manuscript *

Provide the (draft) title of your manuscript.

Chatbot-delivered stage of change tailored web-based intervention to promote physical activity among inactive community-dwelling people aged 65 years or above: protocol of a randomized controlled trial

Name of your App/Software/Intervention *

If there is a short and a long/alternate name, write the short name first and add the long name in brackets.

Web-based interventions delivered by a Chatbc

Evaluated Version (if any)

e.g. "V1", "Release 2017-03-01", "Version 2.0.27913"

Your answer

Language(s) *

What language is the intervention/app in? If multiple languages are available, separate by comma (e.g. "English, French")

Traditional Chinese

URL of your Intervention Website or App

e.g. a direct link to the mobile app on app in appstore (itunes, Google Play), or URL of the website. If the intervention is a DVD or hardware, you can also link to an Amazon page.

Your answer

URL of an image/screenshot (optional)

Your answer

Accessibility *

Can an enduser access the intervention presently?

- ○ access is free and open
- ◉ access only for special usergroups, not open
- ○ access is open to everyone, but requires payment/subscription/in-app purchases
- ○ app/intervention no longer accessible
- ○ Other:

Primary Medical Indication/Disease/Condition *

e.g. "Stress", "Diabetes", or define the target group in brackets after the condition, e.g. "Autism (Parents of children with)", "Alzheimers (Informal Caregivers of)"

Physical activity

Primary Outcomes measured in trial *

comma-separated list of primary outcomes reported in the trial

Physical activity level

Secondary/other outcomes

Are there any other outcomes the intervention is expected to affect?

Your answer

Recommended "Dose" *

What do the instructions for users say on how often the app should be used?

- ○ Approximately Daily
- ◉ Approximately Weekly
- ○ Approximately Monthly
- ○ Approximately Yearly
- ○ "as needed"
- ○ Other:

Approx. Percentage of Users (starters) still using the app as recommended after 3 months *

- ◉ unknown / not evaluated
- ○ 0-10%
- ○ 11-20%
- ○ 21-30%
- ○ 31-40%
- ○ 41-50%
- ○ 51-60%
- ○ 61-70%
- ○ 71%-80%
- ○ 81-90%
- ○ 91-100%
- ○ Other:

Overall, was the app/intervention effective? *

- ◯ yes: all primary outcomes were significantly better in intervention group vs control
- ◯ partly: SOME primary outcomes were significantly better in intervention group vs control
- ◯ no statistically significant difference between control and intervention
- ◯ potentially harmful: control was significantly better than intervention in one or more outcomes
- ◯ inconclusive: more research is needed
- ◉ Other: The study is still ongoing. Data on the comparison of primary outcom

Article Preparation Status/Stage *

At which stage in your article preparation are you currently (at the time you fill in this form)

- ◯ not submitted yet - in early draft status
- ◯ not submitted yet - in late draft status, just before submission
- ◯ submitted to a journal but not reviewed yet
- ◉ submitted to a journal and after receiving initial reviewer comments
- ◯ submitted to a journal and accepted, but not published yet
- ◯ published
- ◯ Other:

Journal *

If you already know where you will submit this paper (or if it is already submitted), please provide the journal name (if it is not JMIR, provide the journal name under "other")

- ◯ not submitted yet / unclear where I will submit this
- ◯ Journal of Medical Internet Research (JMIR)
- ◯ JMIR mHealth and UHealth
- ◯ JMIR Serious Games
- ◯ JMIR Mental Health
- ◯ JMIR Public Health
- ◯ JMIR Formative Research
- ◯ Other JMIR sister journal
- ◉ Other: JMIR Research Protocol

Is this a full powered effectiveness trial or a pilot/feasibility trial? *

- ◯ Pilot/feasibility
- ◉ Fully powered

Manuscript tracking number *

If this is a JMIR submission, please provide the manuscript tracking number under "other" (The ms tracking number can be found in the submission acknowledgement email, or when you login as author in JMIR. If the paper is already published in JMIR, then the ms tracking number is the four-digit number at the end of the DOI, to be found at the bottom of each published article in JMIR)

- ◯ no ms number (yet) / not (yet) submitted to / published in JMIR
- ◉ Other: ms #68796

TITLE AND ABSTRACT

1a) TITLE: Identification as a randomized trial in the title

1a) Does your paper address CONSORT item 1a? *

I.e does the title contain the phrase "Randomized Controlled Trial"? (if not, explain the reason under "other")

- (●) yes
- ( ) Other:

1a-i) Identify the mode of delivery in the title

Identify the mode of delivery. Preferably use “web-based” and/or “mobile” and/or “electronic game” in the title. Avoid ambiguous terms like “online”, “virtual”, “interactive”. Use “Internet-based” only if Intervention includes non-web-based Internet components (e.g. email), use “computer-based” or “electronic” only if offline products are used. Use “virtual” only in the context of “virtual reality” (3-D worlds). Use “online” only in the context of “online support groups”. Complement or substitute product names with broader terms for the class of products (such as “mobile” or “smart phone” instead of “iphone”), especially if the application runs on different platforms.

	1	2	3	4	5	
subitem not at all important	○	○	○	○	○	essential

Does your paper address subitem 1a-i? *

Copy and paste relevant sections from manuscript title (include quotes in quotation marks "like this" to indicate direct quotes from your manuscript), or elaborate on this item by providing additional information not in the ms, or briefly explain why the item is not applicable/relevant for your study

Yes, the title identify the mode of delivery: "Chatbot-delivered stage of change tailored web-based intervention".

1a-ii) Non-web-based components or important co-interventions in title

Mention non-web-based components or important co-interventions in title, if any (e.g., “with telephone support”).

	1	2	3	4	5	
subitem not at all important	○	○	○	○	○	essential

Does your paper address subitem 1a-ii?

Copy and paste relevant sections from manuscript title (include quotes in quotation marks "like this" to indicate direct quotes from your manuscript), or elaborate on this item by providing additional information not in the ms, or briefly explain why the item is not applicable/relevant for your study

The present study does not contain non-web-based components or other important co-interventions

1a-iii) Primary condition or target group in the title

Mention primary condition or target group in the title, if any (e.g., “for children with Type I Diabetes”) Example: A Web-based and Mobile Intervention with Telephone Support for Children with Type I Diabetes: Randomized Controlled Trial

	1	2	3	4	5	
subitem not at all important	○	○	○	○	○	essential

Does your paper address subitem 1a-iii? *

Copy and paste relevant sections from manuscript title (include quotes in quotation marks "like this" to indicate direct quotes from your manuscript), or elaborate on this item by providing additional information not in the ms, or briefly explain why the item is not applicable/relevant for your study

Yes, the title include the target group "inactive community-dwelling people aged 65 years or above"

1b) ABSTRACT: Structured summary of trial design, methods, results, and conclusions

NPT extension: Description of experimental treatment, comparator, care providers, centers, and blinding status.

1b-i) Key features/functionalities/components of the intervention and comparator in the METHODS section of the ABSTRACT

Mention key features/functionalities/components of the intervention and comparator in the abstract. If possible, also mention theories and principles used for designing the site. Keep in mind the needs of systematic reviewers and indexers by including important synonyms. (Note: Only report in the abstract what the main paper is reporting. If this information is missing from the main body of text, consider adding it)

	1	2	3	4	5	
subitem not at all important	○	○	○	○	○	essential

Does your paper address subitem 1b-i? *

Copy and paste relevant sections from the manuscript abstract (include quotes in quotation marks "like this" to indicate direct quotes from your manuscript), or elaborate on this item by providing additional information not in the ms, or briefly explain why the item is not applicable/relevant for your study

Yes, the abstract included the key features of both the intervention and the control conditions: "In the intervention group, Chatbot will measure participants' SOC related to PA, and deliver web-based interventions tailored to their current SOC every week for 12 weeks. In the control group, Chatbot will not measure participants' SOC. It will deliver a standard and non-SOC-tailored web-based intervention every week for 12 weeks."

1b-ii) Level of human involvement in the METHODS section of the ABSTRACT

Clarify the level of human involvement in the abstract, e.g., use phrases like “fully automated” vs. “therapist/nurse/care provider/physician-assisted” (mention number and expertise of providers involved, if any). (Note: Only report in the abstract what the main paper is reporting. If this information is missing from the main body of text, consider adding it)

	1	2	3	4	5	
subitem not at all important	○	○	○	○	○	essential

Does your paper address subitem 1b-ii?

Copy and paste relevant sections from the manuscript abstract (include quotes in quotation marks "like this" to indicate direct quotes from your manuscript), or elaborate on this item by providing additional information not in the ms, or briefly explain why the item is not applicable/relevant for your study

We mentioned "fully-automated" in the abstract

1b-iii) Open vs. closed, web-based (self-assessment) vs. face-to-face assessments in the METHODS section of the ABSTRACT

Mention how participants were recruited (online vs. offline), e.g., from an open access website or from a clinic or a closed online user group (closed usergroup trial), and clarify if this was a purely web-based trial, or there were face-to-face components (as part of the intervention or for assessment). Clearly say if outcomes were self-assessed through questionnaires (as common in web-based trials). Note: In traditional offline trials, an open trial (open-label trial) is a type of clinical trial in which both the researchers and participants know which treatment is being administered. To avoid confusion, use “blinded” or “unblinded” to indicated the level of blinding instead of “open”, as “open” in web-based trials usually refers to “open access” (i.e. participants can self-enrol). (Note: Only report in the abstract what the main paper is reporting. If this information is missing from the main body of text, consider adding it)

	1	2	3	4	5	
subitem not at all important	○	○	○	○	○	essential

Does your paper address subitem 1b-iii?

Copy and paste relevant sections from the manuscript abstract (include quotes in quotation marks "like this" to indicate direct quotes from your manuscript), or elaborate on this item by providing additional information not in the ms, or briefly explain why the item is not applicable/relevant for your study

Your answer

1b-iv) RESULTS section in abstract must contain use data

Report number of participants enrolled/assessed in each group, the use/uptake of the intervention (e.g., attrition/adherence metrics, use over time, number of logins etc.), in addition to primary/secondary outcomes. (Note: Only report in the abstract what the main paper is reporting. If this information is missing from the main body of text, consider adding it)

	1	2	3	4	5	
subitem not at all important	○	○	○	○	○	essential

Does your paper address subitem 1b-iv?

Copy and paste relevant sections from the manuscript abstract (include quotes in quotation marks "like this" to indicate direct quotes from your manuscript), or elaborate on this item by providing additional information not in the ms, or briefly explain why the item is not applicable/relevant for your study

Not applicable. This manuscript is a research protocol. Results are not available at this stage.

1b-v) CONCLUSIONS/DISCUSSION in abstract for negative trials

Conclusions/Discussions in abstract for negative trials: Discuss the primary outcome - if the trial is negative (primary outcome not changed), and the intervention was not used, discuss whether negative results are attributable to lack of uptake and discuss reasons. (Note: Only report in the abstract what the main paper is reporting. If this information is missing from the main body of text, consider adding it)

	1	2	3	4	5	
subitem not at all important	○	○	○	○	○	essential

Does your paper address subitem 1b-v?

Copy and paste relevant sections from the manuscript abstract (include quotes in quotation marks "like this" to indicate direct quotes from your manuscript), or elaborate on this item by providing additional information not in the ms, or briefly explain why the item is not applicable/relevant for your study

Not applicable. This manuscript is a research protocol. Results are not available at this stage.

INTRODUCTION

2a) In INTRODUCTION: Scientific background and explanation of rationale

2a-i) Problem and the type of system/solution

Describe the problem and the type of system/solution that is object of the study: intended as stand-alone intervention vs. incorporated in broader health care program? Intended for a particular patient population? Goals of the intervention, e.g., being more cost-effective to other interventions, replace or complement other solutions? (Note: Details about the intervention are provided in “Methods” under 5)

	1	2	3	4	5	
subitem not at all important	○	○	○	○	○	essential

Does your paper address subitem 2a-i? *

Copy and paste relevant sections from the manuscript (include quotes in quotation marks "like this" to indicate direct quotes from your manuscript), or elaborate on this item by providing additional information not in the ms, or briefly explain why the item is not applicable/relevant for your study

“A number of interventions were conducted to promote PA among older adults. Majority of them used face-to-face approaches such as motivational interviewing, PA coaching, and educational sessions [11]. As compared to no or minimum interventions, these approaches increased objectively measured PA level in both short-term (≤3 months) and mid-term (3-12 months) [11]. However, these interventions required significant manpower and resource to implement. There is an increasing number of web-based interventions for older adults. A systematic review of 18 randomized controlled trials (RCT) showed that web-based interventions were feasible to promote PA among older adults [2]. Digital PA tracking and feedbacks (i.e., providing automatic tracking and feedback on PA level based on pedometers/accelerometers/smartphones) and digital PA coaching (i.e., goal setting, promoting PA according to participants’ baseline PA situations) were most commonly used strategies in these RCT [2]. However, seven of these RCT were feasibility trials pilot studies with limited sample size (10-30 per group) instead of full-powered RCTs, and eleven of them only measured short-term efficacy (≤3 months). Moreover, none of these RCT was conducted in Chinese population.

As the most commonly used stage model, the stage of change (SOC) postulates that completed behavioural change will progress through five ordinal stages (pre-contemplation stage, contemplation stage, preparation stage, action stage and maintenance stage), and individuals require interventions tailored to their current SOC in order to effectively facilitate progression toward sustained behavioural changes [12]. Meta-analysis showed that SOC-tailored interventions are more effective than non-SOC-tailored ones [13]. The SOC has been widely applied to guide the PA promotion in different populations [14-23], including older adults [19,20,22,23]. However, most of the SOC-tailored PA interventions were using face-to-face approaches (e.g., counselling, written material, on-site educational sessions, and motivational interviews) [14-16,18,19,21,23]. It is resources demanding as project staff need to measure the SOC of each participant, and prepare and select interventions tailored to his or her current SOC. To our knowledge, only two web-based PA intervention targeting older adults tried to send some simple tips in text messages tailored to their current SOC [20,22].

Chatbots are computerized programs that replicate human interaction through various forms of communication [24], and they have become increasingly popular in health communication. Systematic reviews highlighted the feasibility and effectiveness of Chatbot in promoting healthy lifestyles, smoking cessation, treatment adherence, vaccination uptake, and reducing substance misuse [25-27]. Two studies applied Chatbot to promote PA among office workers in South Korea [28] and adults in the United States [29]. In the South Korean study, a Chatbot automatically sent a message to participants every day to remind them to perform the stair-climbing behaviours. Once the participants completed the desired behaviour and notified the Chatbot, the Chatbot would automatically provide positive feedbacks to the participants. In addition, coffee coupons were given to those who completed such behaviour. These interventions were shown to be effective in increasing participants’ stair-climbing behaviour [28]. The other study conducted in the United States used a Chatbot to provide knowledge and counselling related to PA, which was effective in increasing participants’ daily steps [29]. Chatbots can proactively interact with the users to determine their SOC and automatically select a pathway of intervention that is tailored to each user’s current SOC, thus making Chatbots ideal for delivering SOC-tailored interventions to promote PA. Previous study demonstrated that applying Chatbot to deliver health promotion was feasible and acceptable among older adults in Hong Kong [30]"

2a-ii) Scientific background, rationale: What is known about the (type of) system

Scientific background, rationale: What is known about the (type of) system that is the object of the study (be sure to discuss the use of similar systems for other conditions/diagnoses, if appropiate), motivation for the study, i.e. what are the reasons for and what is the context for this specific study, from which stakeholder viewpoint is the study performed, potential impact of findings [2]. Briefly justify the choice of the comparator.

	1	2	3	4	5	
subitem not at all important	○	○	○	○	○	essential

Does your paper address subitem 2a-ii? *

Copy and paste relevant sections from the manuscript (include quotes in quotation marks "like this" to indicate direct quotes from your manuscript), or elaborate on this item by providing additional information not in the ms, or briefly explain why the item is not applicable/relevant for your study

"Chatbots are computerized programs that replicate human interaction through various forms of communication [24], and they have become increasingly popular in health communication. Systematic reviews highlighted the feasibility and effectiveness of Chatbot in promoting healthy lifestyles, smoking cessation, treatment adherence, vaccination uptake, and reducing substance misuse [25-27]. Two studies applied Chatbot to promote PA among office workers in South Korea [28] and adults in the United States [29]. In the South Korean study, a Chatbot automatically sent a message to participants every day to remind them to perform the stair-climbing behaviours. Once the participants completed the desired behaviour and notified the Chatbot, the Chatbot would automatically provide positive feedbacks to the participants. In addition, coffee coupons were given to those who completed such behaviour. These interventions were shown to be effective in increasing participants' stair-climbing behaviour [28]. The other study conducted in the United States used a Chatbot to provide knowledge and counselling related to PA, which was effective in increasing participants' daily steps [29]. Chatbots can proactively interact with the users to determine their SOC and automatically select a pathway of intervention that is tailored to each user's current SOC, thus making Chatbots ideal for delivering SOC-tailored interventions to promote PA. Previous study demonstrated that applying Chatbot to deliver health promotion was feasible and acceptable among older adults in Hong Kong [30]"

2b) In INTRODUCTION: Specific objectives or hypotheses

Does your paper address CONSORT subitem 2b? *

Copy and paste relevant sections from the manuscript (include quotes in quotation marks "like this" to indicate direct quotes from your manuscript), or elaborate on this item by providing additional information not in the ms, or briefly explain why the item is not applicable/relevant for your study

"The proposed RCT will compare the efficacy of a SOC-tailored web-based intervention versus a non-SOC-tailored web-based intervention in increasing the prevalence of meeting WHO recommended PA level six months after completion of the intervention among inactive community-dwelling individuals aged ≥65 years.

Secondary objectives of this RCT are to compare the following outcomes six months after completion of the intervention between the intervention and the control group, including: 1) minutes of MPVA, low-intensity PA, and sedentary time in the past week measured by both IPAQ-SF and the accelerometers, 2) step counts in the past week measured by accelerometers, 3) SOC, perceived pros, perceived cons, and perceived self-efficacy related to PA, 4) compliance to the web-based interventions, and 5) cognitive status."

METHODS

3a) Description of trial design (such as parallel, factorial) including allocation ratio

Does your paper address CONSORT subitem 3a? *

Copy and paste relevant sections from the manuscript (include quotes in quotation marks "like this" to indicate direct quotes from your manuscript), or elaborate on this item by providing additional information not in the ms, or briefly explain why the item is not applicable/relevant for your study

Yes. We described the trial design at the beginning of the methods:
"This is a partially blinded (outcome accessors and data analysts) and parallel-group RCT to be conducted in Hong Kong, China. Inactive community-dwelling people aged 65 years or above will be randomized evenly (1:1) into either the intervention group or the control group. In the intervention group, the Chatbot will measure participants' SOC related to PA and deliver web-based interventions tailored to their current SOC every week for 12 weeks. In the control group, the Chatbot will not measure participants' SOC. It will deliver a standard and non-SOC-tailored web-based intervention every week for 12 weeks."

3b) Important changes to methods after trial commencement (such as eligibility criteria), with reasons

Does your paper address CONSORT subitem 3b? *

Copy and paste relevant sections from the manuscript (include quotes in quotation marks "like this" to indicate direct quotes from your manuscript), or elaborate on this item by providing additional information not in the ms, or briefly explain why the item is not applicable/relevant for your study

We did not have important changes after trial commencement.

3b-i) Bug fixes, Downtimes, Content Changes

Bug fixes, Downtimes, Content Changes: ehealth systems are often dynamic systems. A description of changes to methods therefore also includes important changes made on the intervention or comparator during the trial (e.g., major bug fixes or changes in the functionality or content) (5-iii) and other “unexpected events” that may have influenced study design such as staff changes, system failures/downtimes, etc. [2].

	1	2	3	4	5	
subitem not at all important	○	○	○	○	○	essential

Does your paper address subitem 3b-i?

Copy and paste relevant sections from the manuscript (include quotes in quotation marks "like this" to indicate direct quotes from your manuscript), or elaborate on this item by providing additional information not in the ms, or briefly explain why the item is not applicable/relevant for your study

Your answer

4a) Eligibility criteria for participants

Does your paper address CONSORT subitem 4a? *

Copy and paste relevant sections from the manuscript (include quotes in quotation marks "like this" to indicate direct quotes from your manuscript), or elaborate on this item by providing additional information not in the ms, or briefly explain why the item is not applicable/relevant for your study

We described details of the eligibility criteria of the participants:
"Inclusion criteria are: 1) aged 65 years or above, 2) having a Hong Kong identity card, 3) Chinese speaking, 4) not meeting the WHO recommended level of MVPA, which is defined as having <150 minutes of moderate PA in the past week. The interviewers will assess participants using the validated Chinese version of IPAQ-SF, 5) willing to be followed by telephone at T1 and T2, 6) having a smartphone, 7) able to send and read text/voice message via smartphone, and 8) willing to wear an accelerometer for a week at T0, T1 and T2. Exclusion criteria include: 1) blindness or deafness, 2) with diagnosis of major psychiatric diseases (schizophrenia or bipolar disorders) or dementia, 3) with a score ≥1 in the Chinese version of the Physical Activity Readiness Questionnaire (PAR-Q), and 4) with a score ≤16 in the validated telephone version of the Cantonese Mini-mental State Examination (T-CMMSE) [32]"

4a-i) Computer / Internet literacy

Computer / Internet literacy is often an implicit “de facto” eligibility criterion - this should be explicitly clarified.

	1	2	3	4	5	
subitem not at all important	○	○	○	○	○	essential

Does your paper address subitem 4a-i?

Copy and paste relevant sections from the manuscript (include quotes in quotation marks "like this" to indicate direct quotes from your manuscript), or elaborate on this item by providing additional information not in the ms, or briefly explain why the item is not applicable/relevant for your study

Your answer

4a-ii) Open vs. closed, web-based vs. face-to-face assessments:

Open vs. closed, web-based vs. face-to-face assessments: Mention how participants were recruited (online vs. offline), e.g., from an open access website or from a clinic, and clarify if this was a purely web-based trial, or there were face-to-face components (as part of the intervention or for assessment), i.e., to what degree got the study team to know the participant. In online-only trials, clarify if participants were quasi-anonymous and whether having multiple identities was possible or whether technical or logistical measures (e.g., cookies, email confirmation, phone calls) were used to detect/prevent these.

	1	2	3	4	5	
subitem not at all important	○	○	○	○	○	essential

Does your paper address subitem 4a-ii? *

Copy and paste relevant sections from the manuscript (include quotes in quotation marks "like this" to indicate direct quotes from your manuscript), or elaborate on this item by providing additional information not in the ms, or briefly explain why the item is not applicable/relevant for your study

We described the details of the assessment in the outcome section:
"After completion of the baseline telephone survey and before the randomization takes place, participants will be invited to visit our research office to collect the accelerometers. On site, participants will be instructed to Participants will be asked to wear the accelerometers on their waist (i.e., above the right hipbone) with an elastic belt for seven days, including 5 weekdays and 2 weekends, to collect objective data of PA levels. Participants will be asked to wear the accelerometers at all time except when they are showering or swimming. In addition, participants will be reminded to maintain their daily routine when they are wearing the accelerometers. Participants will visit our research office again one week afterwards to return the accelerometers and recall their PA level in the past week by completing the Chinese version of IPAQ-SF. Similar procedures for collecting and returning accelerometers and completing the IPAQ-SF will be arranged after completion of the telephone survey at T1 and T2."

4a-iii) Information giving during recruitment

Information given during recruitment. Specify how participants were briefed for recruitment and in the informed consent procedures (e.g., publish the informed consent documentation as appendix, see also item X26), as this information may have an effect on user self-selection, user expectation and may also bias results.

	1	2	3	4	5	
subitem not at all important	○	○	○	○	○	essential

Does your paper address subitem 4a-iii?

Copy and paste relevant sections from the manuscript (include quotes in quotation marks "like this" to indicate direct quotes from your manuscript), or elaborate on this item by providing additional information not in the ms, or briefly explain why the item is not applicable/relevant for your study

Yes, details were provided:
"Participants will be recruited through random telephone calls. Telephone numbers will be selected from up-to-date Hong Kong telephone directories. Trained interviewers will conduct the telephone calls. If there is more than one person in the household who is aged ≥65 years, the one whose birthday is closest to the interview date will be invited for the study to avoid contamination and introduction of extra confounding factors. Interviewers will brief prospective participants about the study and screen their eligibility."

4b) Settings and locations where the data were collected

Does your paper address CONSORT subitem 4b? *

Copy and paste relevant sections from the manuscript (include quotes in quotation marks "like this" to indicate direct quotes from your manuscript), or elaborate on this item by providing additional information not in the ms, or briefly explain why the item is not applicable/relevant for your study

All interventions of this study were web-based.
This study was conducted in Hong Kong "This is a partially blinded (outcome accessors and data analysts) and parallel-group RCT to be conducted in Hong Kong, China."

4b-i) Report if outcomes were (self-)assessed through online questionnaires

Clearly report if outcomes were (self-)assessed through online questionnaires (as common in web-based trials) or otherwise.

	1	2	3	4	5	
subitem not at all important	○	○	○	○	○	essential

Does your paper address subitem 4b-i? *

Copy and paste relevant sections from the manuscript (include quotes in quotation marks "like this" to indicate direct quotes from your manuscript), or elaborate on this item by providing additional information not in the ms, or briefly explain why the item is not applicable/relevant for your study

The outcomes are measured through telephone interviews and accelerometers. This item is not applicable to this study.

4b-ii) Report how institutional affiliations are displayed

Report how institutional affiliations are displayed to potential participants [on ehealth media], as affiliations with prestigious hospitals or universities may affect volunteer rates, use, and reactions with regards to an intervention.(Not a required item – describe only if this may bias results)

	1	2	3	4	5	
subitem not at all important	○	○	○	○	○	essential

Does your paper address subitem 4b-ii?

Copy and paste relevant sections from the manuscript (include quotes in quotation marks "like this" to indicate direct quotes from your manuscript), or elaborate on this item by providing additional information not in the ms, or briefly explain why the item is not applicable/relevant for your study

Your answer

5) The interventions for each group with sufficient details to allow replication, including how and when they were actually administered

5-i) Mention names, credential, affiliations of the developers, sponsors, and owners

Mention names, credential, affiliations of the developers, sponsors, and owners [6] (if authors/evaluators are owners or developer of the software, this needs to be declared in a “Conflict of interest” section or mentioned elsewhere in the manuscript).

	1	2	3	4	5	
subitem not at all important	○	○	○	○	○	essential

Does your paper address subitem 5-i?

Copy and paste relevant sections from the manuscript (include quotes in quotation marks "like this" to indicate direct quotes from your manuscript), or elaborate on this item by providing additional information not in the ms, or briefly explain why the item is not applicable/relevant for your study

Your answer

5-ii) Describe the history/development process

Describe the history/development process of the application and previous formative evaluations (e.g., focus groups, usability testing), as these will have an impact on adoption/use rates and help with interpreting results.

	1	2	3	4	5	
subitem not at all important	○	○	○	○	○	essential

Does your paper address subitem 5-ii?

Copy and paste relevant sections from the manuscript (include quotes in quotation marks "like this" to indicate direct quotes from your manuscript), or elaborate on this item by providing additional information not in the ms, or briefly explain why the item is not applicable/relevant for your study

The development of the Chatbot was described in the protocol:
"A Chatbot will be developed and maintained on the WhatsApp platform for their wide popularity in local older adults and user-friendly interface for extensions. The architecture of the Chatbot system is shown in Figure 4. Users send information to WhatsApp instant messaging server, which than deliver these messages to the Chatbot. The Chatbot system processes and sends a message back to the WhatsApp, where users can view the information. The knowledge graph, which links participants' input with Chatbot's responses, will be prepared by focus groups of 30 older adults and interviews with expert in sports medicine. The knowledge graph will evolve over time with an increasing number of human-Chatbot interactions. The Chatbot will use a mixture of techniques so that the human-Chatbot interactions will not merely be rule based, but the Chatbot will also learn from the evolving knowledge graph through machine learning. We will pilot the Chatbot among another 30 older adults to improve its accuracy of human-Chatbot interactions. These measures allow the Chatbot have adequate accuracy and consistency in delivering interventions according to the protocol, and avoid the Chatbot going repetitive and off-topic after extensive usage. The same professional team will maintain the health status of the Chatbot during the intervention period. Monthly review of the Chatbot performance will be conducted."

5-iii) Revisions and updating

Revisions and updating. Clearly mention the date and/or version number of the application/intervention (and comparator, if applicable) evaluated, or describe whether the intervention underwent major changes during the evaluation process, or whether the development and/or content was "frozen" during the trial. Describe dynamic components such as news feeds or changing content which may have an impact on the replicability of the intervention (for unexpected events see item 3b).

	1	2	3	4	5	
subitem not at all important	○	○	○	○	○	essential

Does your paper address subitem 5-iii?

Copy and paste relevant sections from the manuscript (include quotes in quotation marks "like this" to indicate direct quotes from your manuscript), or elaborate on this item by providing additional information not in the ms, or briefly explain why the item is not applicable/relevant for your study

Your answer

5-iv) Quality assurance methods

Provide information on quality assurance methods to ensure accuracy and quality of information provided [1], if applicable.

	1	2	3	4	5	
subitem not at all important	○	○	○	○	○	essential

Does your paper address subitem 5-iv?

Copy and paste relevant sections from the manuscript (include quotes in quotation marks "like this" to indicate direct quotes from your manuscript), or elaborate on this item by providing additional information not in the ms, or briefly explain why the item is not applicable/relevant for your study

Your answer

5-v) Ensure replicability by publishing the source code, and/or providing screenshots/screen-capture video, and/or providing flowcharts of the algorithms used

Ensure replicability by publishing the source code, and/or providing screenshots/screen-capture video, and/or providing flowcharts of the algorithms used. Replicability (i.e., other researchers should in principle be able to replicate the study) is a hallmark of scientific reporting.

	1	2	3	4	5	
subitem not at all important	○	○	○	○	○	essential

Does your paper address subitem 5-v?

Copy and paste relevant sections from the manuscript (include quotes in quotation marks "like this" to indicate direct quotes from your manuscript), or elaborate on this item by providing additional information not in the ms, or briefly explain why the item is not applicable/relevant for your study

Your answer

5-vi) Digital preservation

Digital preservation: Provide the URL of the application, but as the intervention is likely to change or disappear over the course of the years; also make sure the intervention is archived (Internet Archive, webcitation.org, and/or publishing the source code or screenshots/videos alongside the article). As pages behind login screens cannot be archived, consider creating demo pages which are accessible without login.

	1	2	3	4	5	
subitem not at all important	○	○	○	○	○	essential

Does your paper address subitem 5-vi?

Copy and paste relevant sections from the manuscript (include quotes in quotation marks "like this" to indicate direct quotes from your manuscript), or elaborate on this item by providing additional information not in the ms, or briefly explain why the item is not applicable/relevant for your study

Your answer

5-vii) Access

Access: Describe how participants accessed the application, in what setting/context, if they had to pay (or were paid) or not, whether they had to be a member of specific group. If known, describe how participants obtained "access to the platform and Internet" [1]. To ensure access for editors/reviewers/readers, consider to provide a "backdoor" login account or demo mode for reviewers/readers to explore the application (also important for archiving purposes, see vi).

	1	2	3	4	5	
subitem not at all important	○	○	○	○	○	essential

Does your paper address subitem 5-vii? *

Copy and paste relevant sections from the manuscript (include quotes in quotation marks "like this" to indicate direct quotes from your manuscript), or elaborate on this item by providing additional information not in the ms, or briefly explain why the item is not applicable/relevant for your study

We described how the users would access the Chatbot:
"Our project staff will connect participants' WhatsApp with the Chatbot, and the Chatbot will automatically run a training module guiding users in an elderly-friendly way when users access it for the first time."

5-viii) Mode of delivery, features/functionalities/components of the intervention and comparator, and the theoretical framework

Describe mode of delivery, features/functionalities/components of the intervention and comparator, and the theoretical framework [6] used to design them (instructional strategy [1], behaviour change techniques, persuasive features, etc., see e.g., [7, 8] for terminology). This includes an in-depth description of the content (including where it is coming from and who developed it) [1]," whether [and how] it is tailored to individual circumstances and allows users to track their progress and receive feedback" [6]. This also includes a description of communication delivery channels and – if computer-mediated communication is a component – whether communication was synchronous or asynchronous [6]. It also includes information on presentation strategies [1], including page design principles, average amount of text on pages, presence of hyperlinks to other resources, etc. [1].

	1	2	3	4	5	
subitem not at all important	○	○	○	○	○	essential

Does your paper address subitem 5-viii? *

Copy and paste relevant sections from the manuscript (include quotes in quotation marks "like this" to indicate direct quotes from your manuscript), or elaborate on this item by providing additional information not in the ms, or briefly explain why the item is not applicable/relevant for your study

We described the details in the protocol:
"The Intervention Group
The interventions start after participants complete baseline PA measurement by the accelerometer. The Chatbot will deliver interventions co-created by researchers and older adults based on the behavioural change strategies recommended by the SOC to address determinants of PA among older adults identified by previous studies. Twelve weekly sessions of SOC-tailored intervention will be delivered by the Chatbot within three months through WhatsApp. In previous studies, 3-month was the most commonly selected intervention duration for web-based interventions promoting PA among older adults [2]. Moreover, one study suggested that some older adults would lose interest if the duration of web-based interventions was longer than three months [33]. Figure 2 shows the workflow of the intervention group. The intervention procedures are described below:
1) Initiate the interventions: the Chatbot greets the participants and states its function and topics to be covered.
2) Measure PA level: the Chatbot asks participants to fill out the Chinese version of IPAQ-SF. The Chatbot will automatically calculate their MVPA level and give feedback based on that level.
3) Identify SOC: the Chatbot asks participants a validated multiple-choice question [33], which has been widely used to identify SOC related to PA: ‘which sentence best describe your current PA situation?’. The response categories are: ‘I participate in PA for less than 150 minutes per week, and do not intend to increase that time in the next six months’ (pre-contemplation stage), ‘I participate in PA for less than 150 minutes per week, and intend to increase that time in the next six months’ (contemplation stage), ‘I participate in PA for less than 150 minutes per week, and intend to increase that time in one month’ (preparation stage), ‘I participate in PA for at least 150 minutes per week, but I have only begun to do so in the last six months’ (action stage), and ‘I participate in PA for at least 150 minutes per week, and have been doing so for more than six months’ (maintenance stage).
4) Deliver interventions tailored to participants’ current SOC: the interventions will address known determinants of PA among older adults identified by the sytematic review and local study [9,10]. Barriers of PA among older adults included lack of knowledge, skills, capacities, or support from peers or family members related to PA, perceived PA would cause pain or injury, difficulties to access sports facilities, and concerns about bad weather, while facilitators included perceived benefits of PA on physical and mental health, perceived self-efficacy to perform PA, and suggestions from health professionals.
i) Pre-contemplation stage: Participants in the pre-contemplation stage will watch a web-based video in which a sport scientist explains the definition of PA, emphasizes adequate levels of MVPA is important for physical and mental health, and introduces levels of MVPA recommended by the WHO for older adults. These contents seek to increase participants’ awareness of the importance of adequate MVPA.
ii) Contemplation stage: Participants in the contemplation stage will watch a different web-based video with the following contents. The same sports scientist talks about the benefits of adequate MVPA for older adults (e.g., reducing dependence-inducing diseases such as cardiovascular diseases and cancers, improve cognitive function, frailty syndrome, performance of physical functions and mental health), addresses physical and environmental barriers of PA by introducing feasible options of PA suitable for older adults that can be performed at home or in the community, and gives recommendation and encourage older adults to make a plan for PA. In addition, several older adults who performed MVPA regularly will share their positive experiences during PA (e.g., better physical functions, stronger immune system, and positive psychological status) and how they overcome physical and environmental barriers of PA. These contents aim to increase self-efficacy and perceived pros and reduce perceived cost of PA.
iii) Preparation stage: the Chatbot provides a dropdown list of easy and enjoyable PA

[illegible]
options that are suitable for older adults, which are identified by focus groups of older adults and suggestions of the sports scientist, to participants in the preparation stage. These options will include brisk walking, aerobics, stretching, strength/muscle training, Tai Chi and eight-section brocade exercise. Participants will be invited to select one or more options from the list. The Chatbot will retrieve pre-designed demonstration video related to each option and send to participants. These contents seek to assist participants in developing and implementing an action plan, and also to increase their perceived self-efficacy related to PA.
iv) Action or maintenance stage: the Chatbot will provide feedback and reinforcement based on their PA level in the previous week. Positive reinforcement is given to participants on track to obtain or have already achieved the WHO-recommended MVPA level. These contents aim to facilitate the maintenance of MVPA and to avoid relapse.
In order to avoid participants watching a same video or reading a same information twice, slightly different videos/information corresponding to each SOC will be prepared. In each session, the Chatbot will randomly select one for the participants. Participants in all stages can select one or more problems about PA from a list of common problems identified by literature review and focus group of older adults. Participants can also raise their problems which are not in the list verbally or in written text. The Chatbot will retrieve relevant information from the knowledge graph and prepare a response.

The Control Group
Firstly, the Chatbot will ask participants to fill out the Chinese version of IPAQ-SF, automatically calculate their MVPA and give feedback based on that level. Then the Chatbot will automatically send a short web-based video covering general information of PA for older adults (e.g., recommended MVPA level, suitable PA options) every week from week 1 to 12. Finally, participants can select problems about PA from the same list in the intervention group. They also have the option to verbally or in written form communicate any issues not included in the list. The Chatbot will extract pertinent information from the same knowledge graph to craft a tailored respons. "

5-ix) Describe use parameters

Describe use parameters (e.g., intended “doses” and optimal timing for use). Clarify what instructions or recommendations were given to the user, e.g., regarding timing, frequency, heaviness of use, if any, or was the intervention used ad libitum.

	1	2	3	4	5	
subitem not at all important	○	○	○	○	○	essential

Does your paper address subitem 5-ix?

Copy and paste relevant sections from the manuscript (include quotes in quotation marks "like this" to indicate direct quotes from your manuscript), or elaborate on this item by providing additional information not in the ms, or briefly explain why the item is not applicable/relevant for your study

Your answer

5-x) Clarify the level of human involvement

Clarify the level of human involvement (care providers or health professionals, also technical assistance) in the e-intervention or as co-intervention (detail number and expertise of professionals involved, if any, as well as "type of assistance offered, the timing and frequency of the support, how it is initiated, and the medium by which the assistance is delivered". It may be necessary to distinguish between the level of human involvement required for the trial, and the level of human involvement required for a routine application outside of a RCT setting (discuss under item 21 – generalizability).

	1	2	3	4	5	
subitem not at all important	○	○	○	○	○	essential

Does your paper address subitem 5-x?

Copy and paste relevant sections from the manuscript (include quotes in quotation marks "like this" to indicate direct quotes from your manuscript), or elaborate on this item by providing additional information not in the ms, or briefly explain why the item is not applicable/relevant for your study

Your answer

5-xi) Report any prompts/reminders used

Report any prompts/reminders used: Clarify if there were prompts (letters, emails, phone calls, SMS) to use the application, what triggered them, frequency etc. It may be necessary to distinguish between the level of prompts/reminders required for the trial, and the level of prompts/reminders for a routine application outside of a RCT setting (discuss under item 21 – generalizability).

	1	2	3	4	5	
subitem not at all important	○	○	○	○	○	essential

Does your paper address subitem 5-xi? *

Copy and paste relevant sections from the manuscript (include quotes in quotation marks "like this" to indicate direct quotes from your manuscript), or elaborate on this item by providing additional information not in the ms, or briefly explain why the item is not applicable/relevant for your study

"If the participants in the intervention group or the control group make no response to the intervention initiated by the Chatbot within 24 hours, the Chatbot will automatically send out four separate reminders afterward. For participants make no response to any reminders, project staff will proactively contact them through WhatsApp/telephone to identify reasons and solve problems."

5-xii) Describe any co-interventions (incl. training/support)

Describe any co-interventions (incl. training/support): Clearly state any interventions that are provided in addition to the targeted eHealth intervention, as ehealth intervention may not be designed as stand-alone intervention. This includes training sessions and support [1]. It may be necessary to distinguish between the level of training required for the trial, and the level of training for a routine application outside of a RCT setting (discuss under item 21 – generalizability.

	1	2	3	4	5	
subitem not at all important	○	○	○	○	○	essential

Does your paper address subitem 5-xii? *

Copy and paste relevant sections from the manuscript (include quotes in quotation marks "like this" to indicate direct quotes from your manuscript), or elaborate on this item by providing additional information not in the ms, or briefly explain why the item is not applicable/relevant for your study

"If the participants in the intervention group or the control group make no response to the intervention initiated by the Chatbot within 24 hours, the Chatbot will automatically send out four separate reminders afterward. For participants make no response to any reminders, project staff will proactively contact them through WhatsApp/telephone to identify reasons and solve problems."

6a) Completely defined pre-specified primary and secondary outcome measures, including how and when they were assessed

Does your paper address CONSORT subitem 6a? *

Copy and paste relevant sections from the manuscript (include quotes in quotation marks "like this" to indicate direct quotes from your manuscript), or elaborate on this item by providing additional information not in the ms, or briefly explain why the item is not applicable/relevant for your study

"Primary Outcome
Prevalence of meeting WHO recommended level of PA (at least 150 minutes moderate-intensity aerobic PA, or at least 75 minutes of vigorous-intensity aerobic PA, or an equivalent combination of MVPA every week) is the primary outcome. PA will be measured by completing the Chinese version of IPAQ-SF and wearing an accelerometer (ActiGraph, Pensacola, FL, USA) at T0, T1, and T2. After completion of the baseline telephone survey and before the randomization takes place, participants will be invited to visit our research office to collect the accelerometers. On site, participants will be instructed to Participants will be asked to wear the accelerometers on their waist (i.e., above the right hipbone) with an elastic belt for seven days, including 5 weekdays and 2 weekends, to collect objective data of PA levels. Participants will be asked to wear the accelerometers at all time except when they are showering or swimming. In addition, participants will be reminded to maintain their daily routine when they are wearing the accelerometers. Participants will visit our research office again one week afterwards to return the accelerometers and recall their PA level in the past week by completing the Chinese version of IPAQ-SF. Similar procedures for collecting and returning accelerometers and completing the IPAQ-SF will be arranged after completion of the telephone survey at T1 and T2. The time on and time off of wearing the accelerometer each day will be recorded, and the data will be used to estimate the time spent in sedentary behaviour, light PA, and MVPA. To ensure the accuracy of data that represent the habitual PA level of participants, data from the participants whose wearing time for less than 600 minutes per day will be considered invalid data. We will calculate sedentary time, light PA, and MVPA using triaxial cut-points. Sedentary time, light PA and MVPA is defined as <200 counts/min, <1951 counts/min, and >1952 counts/min, respectively, with the epoch length being 60s [35].

Secondary Outcomes
The following secondary outcomes will be measured at T0, T1, and T2, including: 1) step counts, and minutes of MVPA, light PA and sedentary time in the last week measured by accelerometer, 2) compliance to the web-based interventions, including number of intervention sessions started and completed by the participants as documented by the Chatbot, 3) SOC related to the PA will be measured by a validated multiple-choice question [34]. The measurement and definition of SOC will be the same as those used by the Chatbot. In addition, we will also extract SOC measured by the Chatbot at the beginning of each intervention session in the intervention group from the chat history. 4) Perceived pros and cons related to PA will be measured by the validated questionnaire on decisional balance developed by Nigg et al, which consists of ten questions with two scales (pros and cons) [36]. 5) Perceived self-efficacy of PA will be measured by a validated 6-item scale developed by Marcus et al [37], and 6) Cognitive status will be measured by the cognitive tasks including sustained attention test [38], working memory [39], inhibitory control [40], and cognitive flexibility [41]."

6a-i) Online questionnaires: describe if they were validated for online use and apply CHERRIES items to describe how the questionnaires were designed/deployed

If outcomes were obtained through online questionnaires, describe if they were validated for online use and apply CHERRIES items to describe how the questionnaires were designed/deployed [9].

	1	2	3	4	5	
subitem not at all important	○	○	○	○	○	essential

Does your paper address subitem 6a-i?

Copy and paste relevant sections from manuscript text

We did not use online questionnaires for assessment.

6a-ii) Describe whether and how “use” (including intensity of use/dosage) was defined/measured/monitored

Describe whether and how “use” (including intensity of use/dosage) was defined/measured/monitored (logins, logfile analysis, etc.). Use/adoption metrics are important process outcomes that should be reported in any ehealth trial.

	1	2	3	4	5	
subitem not at all important	○	○	○	○	○	essential

Does your paper address subitem 6a-ii?

Copy and paste relevant sections from manuscript text

Your answer

6a-iii) Describe whether, how, and when qualitative feedback from participants was obtained

Describe whether, how, and when qualitative feedback from participants was obtained (e.g., through emails, feedback forms, interviews, focus groups).

	1	2	3	4	5	
subitem not at all important	○	○	○	○	○	essential

Does your paper address subitem 6a-iii?

Copy and paste relevant sections from manuscript text

Your answer

6b) Any changes to trial outcomes after the trial commenced, with reasons

Does your paper address CONSORT subitem 6b? *

Copy and paste relevant sections from the manuscript (include quotes in quotation marks "like this" to indicate direct quotes from your manuscript), or elaborate on this item by providing additional information not in the ms, or briefly explain why the item is not applicable/relevant for your study

We did not have any changes to trial outcomes after the trial commenced.

7a) How sample size was determined

NPT: When applicable, details of whether and how the clustering by care provides or centers was addressed

7a-i) Describe whether and how expected attrition was taken into account when calculating the sample size

Describe whether and how expected attrition was taken into account when calculating the sample size.

	1	2	3	4	5	
subitem not at all important	○	○	○	○	○	essential

Does your paper address subitem 7a-i?

Copy and paste relevant sections from manuscript title (include quotes in quotation marks "like this" to indicate direct quotes from your manuscript), or elaborate on this item by providing additional information not in the ms, or briefly explain why the item is not applicable/relevant for your study

"At T0, no participant meets the WHO recommended PA level according to the inclusion criteria. We assume that 10% of participants in the control group will meet the WHO recommended PA level at T2 as there are minimum interventions. A target sample size of 97 per group (194 in total) would allow us to detect a between-group difference of 15% in the prevalence of meeting the WHO recommended PA level at T2 (25% versus 10%) (power: 0.80, alpha: 0.05, NCSS PASS 11.0). We conservatively estimate the dropout rate to be 30% at T2. Thus, 139 eligible participants per group (278 in total) are required."

7b) When applicable, explanation of any interim analyses and stopping guidelines

Does your paper address CONSORT subitem 7b? *

Copy and paste relevant sections from the manuscript (include quotes in quotation marks "like this" to indicate direct quotes from your manuscript), or elaborate on this item by providing additional information not in the ms, or briefly explain why the item is not applicable/relevant for your study

We did not have any interim analyses or stopping guidelines for this RCT.

8a) Method used to generate the random allocation sequence

NPT: When applicable, how care providers were allocated to each trial group

Does your paper address CONSORT subitem 8a? *

Copy and paste relevant sections from the manuscript (include quotes in quotation marks "like this" to indicate direct quotes from your manuscript), or elaborate on this item by providing additional information not in the ms, or briefly explain why the item is not applicable/relevant for your study

"Computer-generated random allocation codes will be produced and sealed in opaque envelopes by a research staff member, without involvement in recruitment or baseline survey. One envelope will be drawn and opened by the interviewer, who then informs the participants about their assigned group. Block randomization with a block size of eight will be used."

8b) Type of randomisation; details of any restriction (such as blocking and block size)

Does your paper address CONSORT subitem 8b? *

Copy and paste relevant sections from the manuscript (include quotes in quotation marks "like this" to indicate direct quotes from your manuscript), or elaborate on this item by providing additional information not in the ms, or briefly explain why the item is not applicable/relevant for your study

"Block randomization with a block size of eight will be used."

9) Mechanism used to implement the random allocation sequence (such as sequentially numbered containers), describing any steps taken to conceal the sequence until interventions were assigned

Does your paper address CONSORT subitem 9? *

Copy and paste relevant sections from the manuscript (include quotes in quotation marks "like this" to indicate direct quotes from your manuscript), or elaborate on this item by providing additional information not in the ms, or briefly explain why the item is not applicable/relevant for your study

"Computer-generated random allocation codes will be produced and sealed in opaque envelopes by a research staff member, without involvement in recruitment or baseline survey. One envelope will be drawn and opened by the interviewer, who then informs the participants about their assigned group."

10) Who generated the random allocation sequence, who enrolled participants, and who assigned participants to interventions

Does your paper address CONSORT subitem 10? *

Copy and paste relevant sections from the manuscript (include quotes in quotation marks "like this" to indicate direct quotes from your manuscript), or elaborate on this item by providing additional information not in the ms, or briefly explain why the item is not applicable/relevant for your study

"Computer-generated random allocation codes will be produced and sealed in opaque envelopes by a research staff member, without involvement in recruitment or baseline survey."

11a) If done, who was blinded after assignment to interventions (for example, participants, care providers, those assessing outcomes) and how

NPT: Whether or not administering co-interventions were blinded to group assignment

11a-i) Specify who was blinded, and who wasn't

Specify who was blinded, and who wasn't. Usually, in web-based trials it is not possible to blind the participants [1, 3] (this should be clearly acknowledged), but it may be possible to blind outcome assessors, those doing data analysis or those administering co-interventions (if any).

	1	2	3	4	5	
subitem not at all important	○	○	○	○	○	essential

Does your paper address subitem 11a-i? *

Copy and paste relevant sections from the manuscript (include quotes in quotation marks "like this" to indicate direct quotes from your manuscript), or elaborate on this item by providing additional information not in the ms, or briefly explain why the item is not applicable/relevant for your study

"The outcome assessors and data analysts will be blinded to the treatment allocation. Participants are reminded not to disclose their received intervention to assessors."

11a-ii) Discuss e.g., whether participants knew which intervention was the "intervention of interest" and which one was the "comparator"

Informed consent procedures (4a-ii) can create biases and certain expectations - discuss e.g., whether participants knew which intervention was the "intervention of interest" and which one was the "comparator".

	1	2	3	4	5	
subitem not at all important	○	○	○	○	○	essential

Does your paper address subitem 11a-ii?

Copy and paste relevant sections from the manuscript (include quotes in quotation marks "like this" to indicate direct quotes from your manuscript), or elaborate on this item by providing additional information not in the ms, or briefly explain why the item is not applicable/relevant for your study

Participants knew the intervention and the control conditions.

11b) If relevant, description of the similarity of interventions

(this item is usually not relevant for ehealth trials as it refers to similarity of a placebo or sham intervention to a active medication/intervention)

Does your paper address CONSORT subitem 11b? *

Copy and paste relevant sections from the manuscript (include quotes in quotation marks "like this" to indicate direct quotes from your manuscript), or elaborate on this item by providing additional information not in the ms, or briefly explain why the item is not applicable/relevant for your study

This is a RCT evaluating ehealth interventions. This item is not relevant to this RCT.

12a) Statistical methods used to compare groups for primary and secondary outcomes

NPT: When applicable, details of whether and how the clustering by care providers or centers was addressed

Does your paper address CONSORT subitem 12a? *

Copy and paste relevant sections from the manuscript (include quotes in quotation marks "like this" to indicate direct quotes from your manuscript), or elaborate on this item by providing additional information not in the ms, or briefly explain why the item is not applicable/relevant for your study

"Intention-to-treat analysis will be performed. Markov Chain Monte Carlo Method will be used to impute missing primary and secondary outcomes. Variables to impute the missing values include participants' background characteristics and baseline values of these outcomes. Chi-square and independent sample t tests are used to examine between-group balance of potential confounders at baseline. We will compare between-group difference in primary and secondary outcomes at both T1 and T2. The relative risk, absolute risk reduction, and number needed to treat and their 95% confidence interval will be calculated by using Excel. Logistic regression for binary variables and linear regression models for continuous variables will be used to test the between-group difference in the primary and secondary outcomes, after controlling for any baseline confounders with $P < 0.20$ in between-group comparisons. SPSS, version 26.0 (IBM SPSS) and R program version 4.3.1 (MO, USA) will be used for data analysis with two-sided with $P < 0.05$ as statistically significant."

12a-i) Imputation techniques to deal with attrition / missing values

Imputation techniques to deal with attrition / missing values: Not all participants will use the intervention/comparator as intended and attrition is typically high in ehealth trials. Specify how participants who did not use the application or dropped out from the trial were treated in the statistical analysis (a complete case analysis is strongly discouraged, and simple imputation techniques such as LOCF may also be problematic [4]).

	1	2	3	4	5	
subitem not at all important	○	○	○	○	○	essential

Does your paper address subitem 12a-i? *

Copy and paste relevant sections from the manuscript (include quotes in quotation marks "like this" to indicate direct quotes from your manuscript), or elaborate on this item by providing additional information not in the ms, or briefly explain why the item is not applicable/relevant for your study

"Markov Chain Monte Carlo Method will be used to impute missing primary and secondary outcomes. Variables to impute the missing values include participants' background characteristics and baseline values of these outcomes."

12b) Methods for additional analyses, such as subgroup analyses and adjusted analyses

Does your paper address CONSORT subitem 12b? *

Copy and paste relevant sections from the manuscript (include quotes in quotation marks "like this" to indicate direct quotes from your manuscript), or elaborate on this item by providing additional information not in the ms, or briefly explain why the item is not applicable/relevant for your study

"Logistic regression for binary variables and linear regression models for continuous variables will be used to test the between-group difference in the primary and secondary outcomes, after controlling for any baseline confounders with P < 0.20 in between-group comparisons."

X26) REB/IRB Approval and Ethical Considerations [recommended as subheading under "Methods"] (not a CONSORT item)

X26-i) Comment on ethics committee approval

	1	2	3	4	5	
subitem not at all important	○	○	○	○	○	essential

Does your paper address subitem X26-i?

Copy and paste relevant sections from the manuscript (include quotes in quotation marks "like this" to indicate direct quotes from your manuscript), or elaborate on this item by providing additional information not in the ms, or briefly explain why the item is not applicable/relevant for your study

"Human subject ethics review approvals
Ethics approval was obtained from the Survey and Behavioural Research Ethics Committee of the Chinese University of Hong Kong (SBRE-22-0257) and the Joint Chinese University of Hong Kong – New Territories East Cluster Clinical Research Ethics Committee (2022.610-T).
"

x26-ii) Outline informed consent procedures

Outline informed consent procedures e.g., if consent was obtained offline or online (how? Checkbox, etc.?), and what information was provided (see 4a-ii). See [6] for some items to be included in informed consent documents.

	1	2	3	4	5	
subitem not at all important	○	○	○	○	○	essential

Does your paper address subitem X26-ii?

Copy and paste relevant sections from the manuscript (include quotes in quotation marks "like this" to indicate direct quotes from your manuscript), or elaborate on this item by providing additional information not in the ms, or briefly explain why the item is not applicable/relevant for your study

"All participants will fill out an electronic consent form through a link sent by WhatsApp to confirm their informed consent."

X26-iii) Safety and security procedures

Safety and security procedures, incl. privacy considerations, and any steps taken to reduce the likelihood or detection of harm (e.g., education and training, availability of a hotline)

	1	2	3	4	5	
subitem not at all important	○	○	○	○	○	essential

Does your paper address subitem X26-iii?

Copy and paste relevant sections from the manuscript (include quotes in quotation marks "like this" to indicate direct quotes from your manuscript), or elaborate on this item by providing additional information not in the ms, or briefly explain why the item is not applicable/relevant for your study

"Participants can report adverse events related to PA through WhatsApp or a hotline during the study period. If sustained serious adverse events are found, advice will be sought from a sport science expert."

RESULTS

13a) For each group, the numbers of participants who were randomly assigned, received intended treatment, and were analysed for the primary outcome

NPT: The number of care providers or centers performing the intervention in each group and the number of patients treated by each care provider in each center

Does your paper address CONSORT subitem 13a? *

Copy and paste relevant sections from the manuscript (include quotes in quotation marks "like this" to indicate direct quotes from your manuscript), or elaborate on this item by providing additional information not in the ms, or briefly explain why the item is not applicable/relevant for your study

"Study recruitment started in November 2024. By the end of February 2025, a total of 185 participants completed the baseline assessment, and were randomly assigned to either the intervention group (n=93) or the control group (n=92). The follow-up assessment at T1 started in March 2025. The, and data collection is expected to be concluded in February 2026. "

13b) For each group, losses and exclusions after randomisation, together with reasons

Does your paper address CONSORT subitem 13b? (NOTE: Preferably, this is shown in a CONSORT flow diagram) *

Copy and paste relevant sections from the manuscript (include quotes in quotation marks "like this" to indicate direct quotes from your manuscript), or elaborate on this item by providing additional information not in the ms, or briefly explain why the item is not applicable/relevant for your study

This is a research protocol. The data on losses and exclusions after randomization is unavailable at this stage.

13b-i) Attrition diagram

Strongly recommended: An attrition diagram (e.g., proportion of participants still logging in or using the intervention/comparator in each group plotted over time, similar to a survival curve) or other figures or tables demonstrating usage/dose/engagement.

	1	2	3	4	5	
subitem not at all important	○	○	○	○	○	essential

Does your paper address subitem 13b-i?

Copy and paste relevant sections from the manuscript or cite the figure number if applicable (include quotes in quotation marks "like this" to indicate direct quotes from your manuscript), or elaborate on this item by providing additional information not in the ms, or briefly explain why the item is not applicable/relevant for your study

This is a research protocol. The data on attrition is unavailable at this stage.

14a) Dates defining the periods of recruitment and follow-up

Does your paper address CONSORT subitem 14a? *

Copy and paste relevant sections from the manuscript (include quotes in quotation marks "like this" to indicate direct quotes from your manuscript), or elaborate on this item by providing additional information not in the ms, or briefly explain why the item is not applicable/relevant for your study

Study recruitment started in November 2024 and is expected to be completed in May 2025.

14a-i) Indicate if critical "secular events" fell into the study period

Indicate if critical "secular events" fell into the study period, e.g., significant changes in Internet resources available or "changes in computer hardware or Internet delivery resources"

	1	2	3	4	5	
subitem not at all important	○	○	○	○	○	essential

Does your paper address subitem 14a-i?

Copy and paste relevant sections from the manuscript (include quotes in quotation marks "like this" to indicate direct quotes from your manuscript), or elaborate on this item by providing additional information not in the ms, or briefly explain why the item is not applicable/relevant for your study

Your answer

14b) Why the trial ended or was stopped (early)

Does your paper address CONSORT subitem 14b? *

Copy and paste relevant sections from the manuscript (include quotes in quotation marks "like this" to indicate direct quotes from your manuscript), or elaborate on this item by providing additional information not in the ms, or briefly explain why the item is not applicable/relevant for your study

This is a research protocol. The RCT is still ongoing.

15) A table showing baseline demographic and clinical characteristics for each group

NPT: When applicable, a description of care providers (case volume, qualification, expertise, etc.) and centers (volume) in each group

Does your paper address CONSORT subitem 15? *

Copy and paste relevant sections from the manuscript (include quotes in quotation marks "like this" to indicate direct quotes from your manuscript), or elaborate on this item by providing additional information not in the ms, or briefly explain why the item is not applicable/relevant for your study

This is a research protocol. The data on baseline characteristics are not available at this stage.

15-i) Report demographics associated with digital divide issues

In ehealth trials it is particularly important to report demographics associated with digital divide issues, such as age, education, gender, social-economic status, computer/Internet/ehealth literacy of the participants, if known.

	1	2	3	4	5	
subitem not at all important	○	○	○	○	○	essential

Does your paper address subitem 15-i? *

Copy and paste relevant sections from the manuscript (include quotes in quotation marks "like this" to indicate direct quotes from your manuscript), or elaborate on this item by providing additional information not in the ms, or briefly explain why the item is not applicable/relevant for your study

This is a research protocol. The data on baseline characteristics are not available at this stage.

16) For each group, number of participants (denominator) included in each analysis and whether the analysis was by original assigned groups

16-i) Report multiple “denominators” and provide definitions

Report multiple “denominators” and provide definitions: Report N’s (and effect sizes) “across a range of study participation [and use] thresholds” [1], e.g., N exposed, N consented, N used more than x times, N used more than y weeks, N participants “used” the intervention/comparator at specific pre-defined time points of interest (in absolute and relative numbers per group). Always clearly define “use” of the intervention.

	1	2	3	4	5	
subitem not at all important	O	O	O	O	O	essential

Does your paper address subitem 16-i? *

Copy and paste relevant sections from the manuscript (include quotes in quotation marks "like this" to indicate direct quotes from your manuscript), or elaborate on this item by providing additional information not in the ms, or briefly explain why the item is not applicable/relevant for your study

This is a research protocol. The data on baseline characteristics are not available at this stage.

16-ii) Primary analysis should be intent-to-treat

Primary analysis should be intent-to-treat, secondary analyses could include comparing only “users”, with the appropriate caveats that this is no longer a randomized sample (see 18-i).

	1	2	3	4	5	
subitem not at all important	○	○	○	○	○	essential

Does your paper address subitem 16-ii?

Copy and paste relevant sections from the manuscript (include quotes in quotation marks "like this" to indicate direct quotes from your manuscript), or elaborate on this item by providing additional information not in the ms, or briefly explain why the item is not applicable/relevant for your study

"Intention-to-treat analysis will be performed."

17a) For each primary and secondary outcome, results for each group, and the estimated effect size and its precision (such as 95% confidence interval)

Does your paper address CONSORT subitem 17a? *

Copy and paste relevant sections from the manuscript (include quotes in quotation marks "like this" to indicate direct quotes from your manuscript), or elaborate on this item by providing additional information not in the ms, or briefly explain why the item is not applicable/relevant for your study

"The relative risk, absolute risk reduction, and number needed to treat and their 95% confidence interval will be calculated by using Excel."

17a-i) Presentation of process outcomes such as metrics of use and intensity of use

In addition to primary/secondary (clinical) outcomes, the presentation of process outcomes such as metrics of use and intensity of use (dose, exposure) and their operational definitions is critical. This does not only refer to metrics of attrition (13-b) (often a binary variable), but also to more continuous exposure metrics such as "average session length". These must be accompanied by a technical description how a metric like a "session" is defined (e.g., timeout after idle time) [1] (report under item 6a).

	1	2	3	4	5	
subitem not at all important	O	O	O	O	O	essential

Does your paper address subitem 17a-i?

Copy and paste relevant sections from the manuscript (include quotes in quotation marks "like this" to indicate direct quotes from your manuscript), or elaborate on this item by providing additional information not in the ms, or briefly explain why the item is not applicable/relevant for your study

Your answer

17b) For binary outcomes, presentation of both absolute and relative effect sizes is recommended

Does your paper address CONSORT subitem 17b? *

Copy and paste relevant sections from the manuscript (include quotes in quotation marks "like this" to indicate direct quotes from your manuscript), or elaborate on this item by providing additional information not in the ms, or briefly explain why the item is not applicable/relevant for your study

"The relative risk, absolute risk reduction, and number needed to treat and their 95% confidence interval will be calculated by using Excel."

18) Results of any other analyses performed, including subgroup analyses and adjusted analyses, distinguishing pre-specified from exploratory

Does your paper address CONSORT subitem 18? *

Copy and paste relevant sections from the manuscript (include quotes in quotation marks "like this" to indicate direct quotes from your manuscript), or elaborate on this item by providing additional information not in the ms, or briefly explain why the item is not applicable/relevant for your study

"Logistic regression for binary variables and linear regression models for continuous variables will be used to test the between-group difference in the primary and secondary outcomes, after controlling for any baseline confounders with P < 0.20 in between-group comparisons."

18-i) Subgroup analysis of comparing only users

A subgroup analysis of comparing only users is not uncommon in ehealth trials, but if done, it must be stressed that this is a self-selected sample and no longer an unbiased sample from a randomized trial (see 16-iii).

	1	2	3	4	5	
subitem not at all important	○	○	○	○	○	essential

Does your paper address subitem 18-i?

Copy and paste relevant sections from the manuscript (include quotes in quotation marks "like this" to indicate direct quotes from your manuscript), or elaborate on this item by providing additional information not in the ms, or briefly explain why the item is not applicable/relevant for your study

Your answer

19) All important harms or unintended effects in each group

(for specific guidance see CONSORT for harms)

Does your paper address CONSORT subitem 19? *

Copy and paste relevant sections from the manuscript (include quotes in quotation marks "like this" to indicate direct quotes from your manuscript), or elaborate on this item by providing additional information not in the ms, or briefly explain why the item is not applicable/relevant for your study

This is a research protocol. The data on unintended harms is not available at this stage.

19-i) Include privacy breaches, technical problems

Include privacy breaches, technical problems. This does not only include physical “harm” to participants, but also incidents such as perceived or real privacy breaches [1], technical problems, and other unexpected/unintended incidents. “Unintended effects” also includes unintended positive effects [2].

	1	2	3	4	5	
subitem not at all important	○	○	○	○	○	essential

Does your paper address subitem 19-i?

Copy and paste relevant sections from the manuscript (include quotes in quotation marks "like this" to indicate direct quotes from your manuscript), or elaborate on this item by providing additional information not in the ms, or briefly explain why the item is not applicable/relevant for your study

Your answer

19-ii) Include qualitative feedback from participants or observations from staff/researchers

Include qualitative feedback from participants or observations from staff/researchers, if available, on strengths and shortcomings of the application, especially if they point to unintended/unexpected effects or uses. This includes (if available) reasons for why people did or did not use the application as intended by the developers.

	1	2	3	4	5	
subitem not at all important	○	○	○	○	○	essential

Does your paper address subitem 19-ii?

Copy and paste relevant sections from the manuscript (include quotes in quotation marks "like this" to indicate direct quotes from your manuscript), or elaborate on this item by providing additional information not in the ms, or briefly explain why the item is not applicable/relevant for your study

Your answer

DISCUSSION

22) Interpretation consistent with results, balancing benefits and harms, and considering other relevant evidence

NPT: In addition, take into account the choice of the comparator, lack of or partial blinding, and unequal expertise of care providers or centers in each group

22-i) Restate study questions and summarize the answers suggested by the data, starting with primary outcomes and process outcomes (use)

Restate study questions and summarize the answers suggested by the data, starting with primary outcomes and process outcomes (use).

	1	2	3	4	5	
subitem not at all important	○	○	○	○	○	essential

Does your paper address subitem 22-i? *

Copy and paste relevant sections from the manuscript (include quotes in quotation marks "like this" to indicate direct quotes from your manuscript), or elaborate on this item by providing additional information not in the ms, or briefly explain why the item is not applicable/relevant for your study

This is a research protocol. This item is not applicable to this manuscript.

22-ii) Highlight unanswered new questions, suggest future research

Highlight unanswered new questions, suggest future research.

	1	2	3	4	5	
subitem not at all important	○	○	○	○	○	essential

Does your paper address subitem 22-ii?

Copy and paste relevant sections from the manuscript (include quotes in quotation marks "like this" to indicate direct quotes from your manuscript), or elaborate on this item by providing additional information not in the ms, or briefly explain why the item is not applicable/relevant for your study

This is a research protocol. This item is not applicable to this manuscript.

20) Trial limitations, addressing sources of potential bias, imprecision, and, if relevant, multiplicity of analyses

20-i) Typical limitations in ehealth trials

Typical limitations in ehealth trials: Participants in ehealth trials are rarely blinded. Ehealth trials often look at a multiplicity of outcomes, increasing risk for a Type I error. Discuss biases due to non-use of the intervention/usability issues, biases through informed consent procedures, unexpected events.

	1	2	3	4	5	
subitem not at all important	○	○	○	○	○	essential

Does your paper address subitem 20-i? *

Copy and paste relevant sections from the manuscript (include quotes in quotation marks "like this" to indicate direct quotes from your manuscript), or elaborate on this item by providing additional information not in the ms, or briefly explain why the item is not applicable/relevant for your study

"This study has some limitations. First, we do not have a control group without suing the Chatbot. This RCT cannot provide evidence to support whether the interventions delivered by the Chatbot will be more effective than the intervention without a Chatbot. Second, the intervention is limited to people aged 65 years or above who have access to smartphones. In Hong Kong, 73% of adults of this age group had smartphones in 2021 [42]. The smartphone ownership has been increasing sharply. Third, participants and those who refuse to participate in this study may have different characteristics and motivation to perform PA. We are not able to collect information from refusals, selection bias exists. Moreover, as compared to other RCTs promoting PA among older adults [2], our follow-up period is shorter (6 months versus 12 months). We selected a shorter follow-up period due to some practical concerns. The effect size of the intervention would be smaller and the dropout rate would be higher if we choose a longer follow-up period. Therefore, we need a much larger sample size, which is not feasible under the current funding scope. The limited ability to observe long-term effects of the interventions due to the relatively short follow-up period is a limitation of this study. Moreover, it is possible that participants' behaviours would be altered while wearing accelerometers. Meta-analysis showed that when an accelerometer was providing real-time monitoring and feedback of PA level, using it alone or together with other interventions would result in improvement in PA among older adults [43]. However, the accelerometer will only be used to record participants' PA levels in this study. It will not give any feedback and the users are not able to access the accelerometer data during the assessment period. Previous study showed that when an accelerometer was only used for outcome assessment, there was no difference in PA level between those who wore accelerometers and those who did not [44]. An accelerometer was commonly used to measure older adults' PA levels in previous RCTs [45]."

21) Generalisability (external validity, applicability) of the trial findings

NPT: External validity of the trial findings according to the intervention, comparators, patients, and care providers or centers involved in the trial

21-i) Generalizability to other populations

Generalizability to other populations: In particular, discuss generalizability to a general Internet population, outside of a RCT setting, and general patient population, including applicability of the study results for other organizations

	1	2	3	4	5	
subitem not at all important	○	○	○	○	○	essential

Does your paper address subitem 21-i?

Copy and paste relevant sections from the manuscript (include quotes in quotation marks "like this" to indicate direct quotes from your manuscript), or elaborate on this item by providing additional information not in the ms, or briefly explain why the item is not applicable/relevant for your study

Your answer

21-ii) Discuss if there were elements in the RCT that would be different in a routine application setting

Discuss if there were elements in the RCT that would be different in a routine application setting (e.g., prompts/reminders, more human involvement, training sessions or other co-interventions) and what impact the omission of these elements could have on use, adoption, or outcomes if the intervention is applied outside of a RCT setting.

	1	2	3	4	5	
subitem not at all important	○	○	○	○	○	essential

Does your paper address subitem 21-ii?

Copy and paste relevant sections from the manuscript (include quotes in quotation marks "like this" to indicate direct quotes from your manuscript), or elaborate on this item by providing additional information not in the ms, or briefly explain why the item is not applicable/relevant for your study

Your answer

OTHER INFORMATION

23) Registration number and name of trial registry

Does your paper address CONSORT subitem 23? *

Copy and paste relevant sections from the manuscript (include quotes in quotation marks "like this" to indicate direct quotes from your manuscript), or elaborate on this item by providing additional information not in the ms, or briefly explain why the item is not applicable/relevant for your study

"The study was registered at ClinicalTrials.gov (NCT06641492)"

24) Where the full trial protocol can be accessed, if available

Does your paper address CONSORT subitem 24? *

Cite a Multimedia Appendix, other reference, or copy and paste relevant sections from the manuscript (include quotes in quotation marks "like this" to indicate direct quotes from your manuscript), or elaborate on this item by providing additional information not in the ms, or briefly explain why the item is not applicable/relevant for your study

This manuscript is the full trial protocol

25) Sources of funding and other support (such as supply of drugs), role of funders

Does your paper address CONSORT subitem 25? *

Copy and paste relevant sections from the manuscript (include quotes in quotation marks "like this" to indicate direct quotes from your manuscript), or elaborate on this item by providing additional information not in the ms, or briefly explain why the item is not applicable/relevant for your study

"This study was supported by the Health and Medical Research Fund, Health Bureau, Hong Kong Special Administrative Region (grant number: 20210531)"

X27) Conflicts of Interest (not a CONSORT item)

X27-i) State the relation of the study team towards the system being evaluated

In addition to the usual declaration of interests (financial or otherwise), also state the relation of the study team towards the system being evaluated, i.e., state if the authors/evaluators are distinct from or identical with the developers/sponsors of the intervention.

	1	2	3	4	5	
subitem not at all important	○	○	○	○	○	essential

Does your paper address subitem X27-i?

Copy and paste relevant sections from the manuscript (include quotes in quotation marks "like this" to indicate direct quotes from your manuscript), or elaborate on this item by providing additional information not in the ms, or briefly explain why the item is not applicable/relevant for your study

Your answer

About the CONSORT EHEALTH checklist

As a result of using this checklist, did you make changes in your manuscript? *

- ○ yes, major changes
- ◉ yes, minor changes
- ○ no

What were the most important changes you made as a result of using this checklist?

Your answer

How much time did you spend on going through the checklist INCLUDING making changes in your manuscript *

I spent about 1 hour going through the checklist.

As a result of using this checklist, do you think your manuscript has improved? *

- (selected) yes
- no
- Other:

Would you like to become involved in the CONSORT EHEALTH group?

This would involve for example becoming involved in participating in a workshop and writing an "Explanation and Elaboration" document

- yes
- no
- Other:

Any other comments or questions on CONSORT EHEALTH

Your answer

STOP - Save this form as PDF before you click submit

To generate a record that you filled in this form, we recommend to generate a PDF of this page (on a Mac, simply select "print" and then select "print as PDF") before you submit it.

When you submit your (revised) paper to JMIR, please upload the PDF as supplementary file.

Don't worry if some text in the textboxes is cut off, as we still have the complete information in our database. Thank you!

Final step: Click submit !

Click submit so we have your answers in our database!

Submit Clear form

Never submit passwords through Google Forms.

This content is neither created nor endorsed by Google. - Terms of Service - Privacy Policy

Does this form look suspicious? Report

Google Forms
