# Supplementary figures and images for: Chatbot-Delivered Stage of Change–Tailored Web-Based Intervention to Promote Physical Activity Among Inactive Community-Dwelling People Aged 65 years or More: Protocol for a Randomized Controlled Trial
Source: JMIR Res Protoc. 2025 Jun 20;14:e68796. doi: 10.2196/68796 (PMC12228010; doi:10.2196/68796)

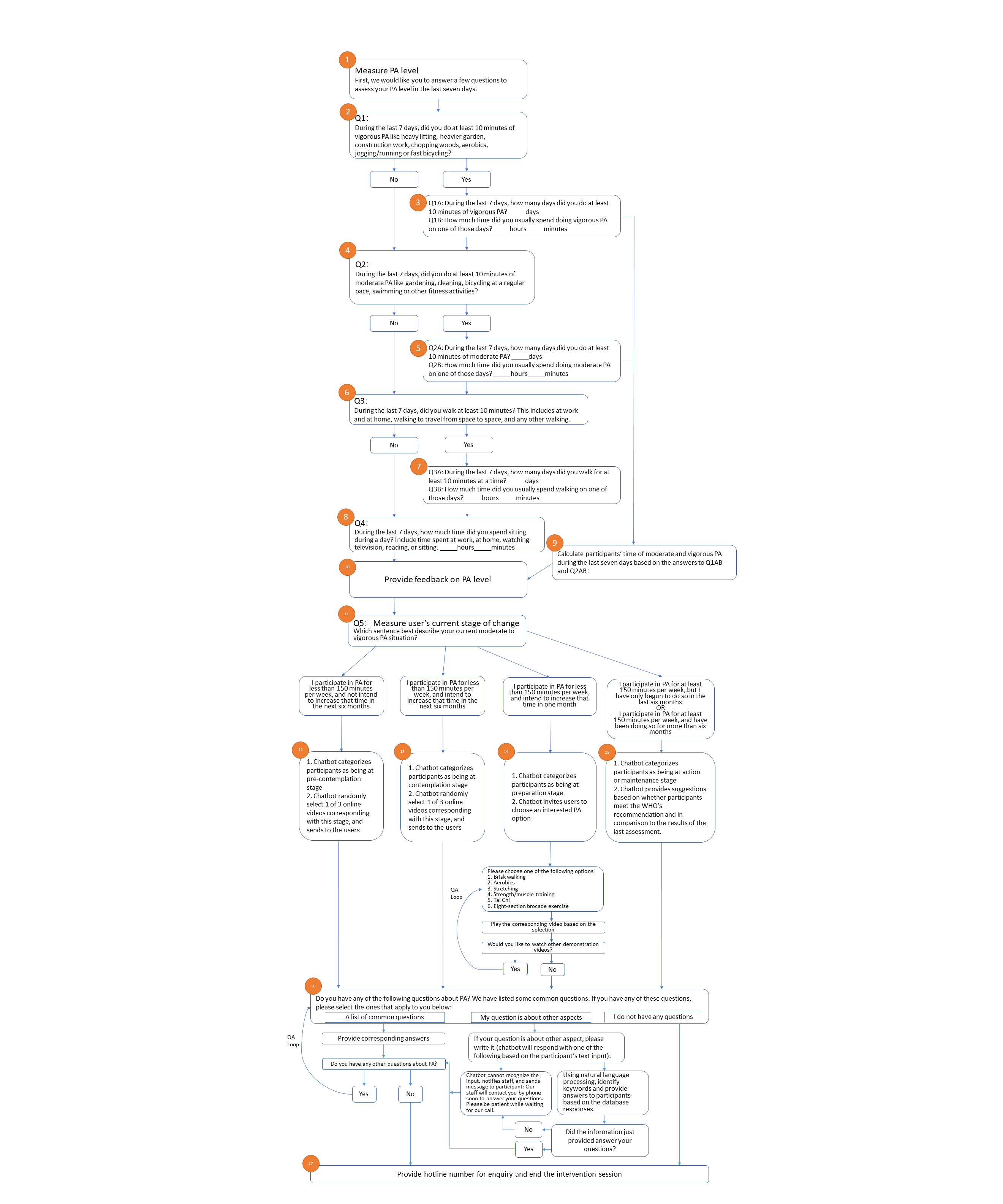

Supplement: Multimedia Appendix 3 [file resprot_v14i1e68796_app3.png]

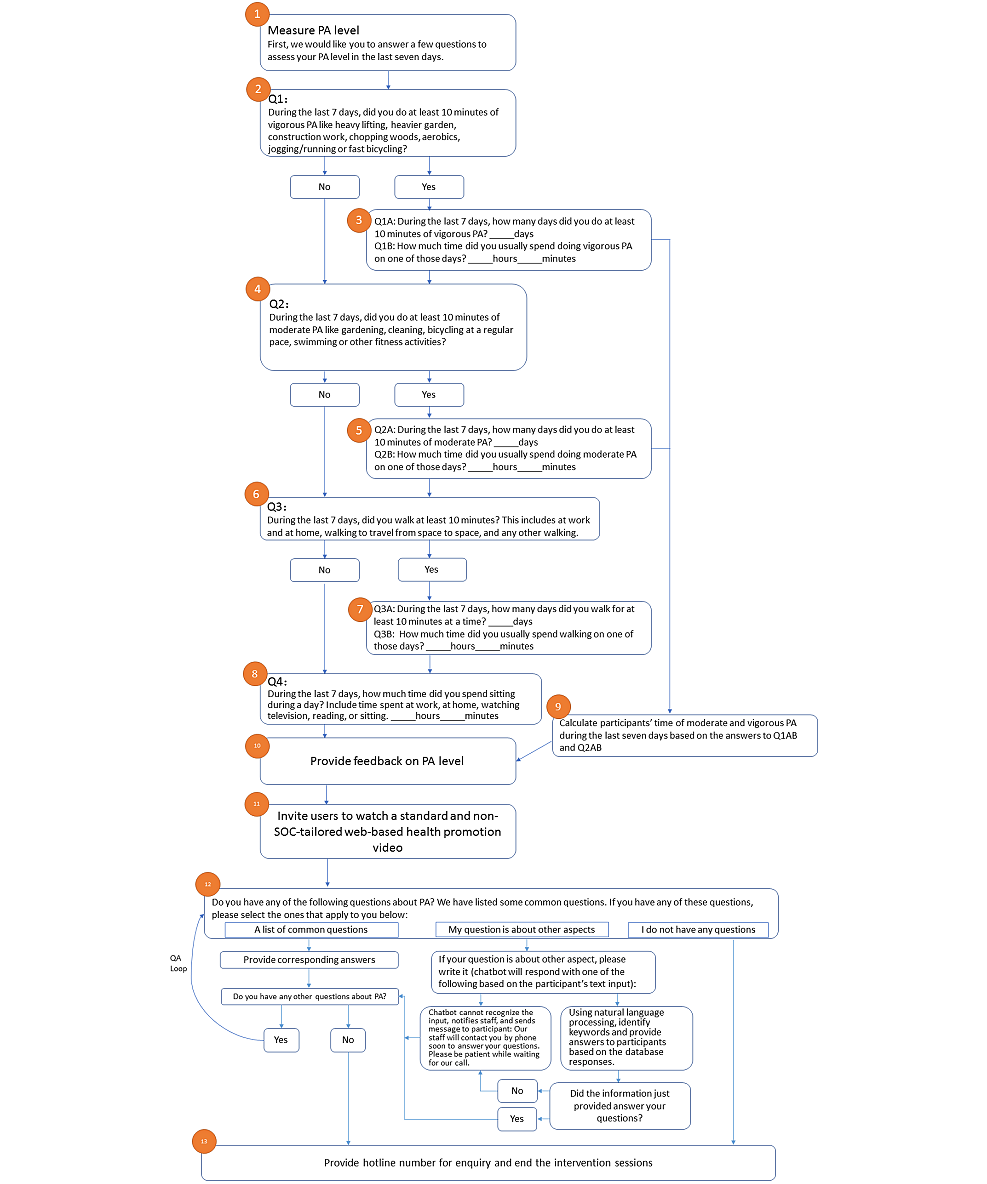

Supplement: Multimedia Appendix 4 [file resprot_v14i1e68796_app4.png]
