# Supplementary material for: Chatbot-Delivered Stage of Change–Tailored Web-Based Intervention to Promote Physical Activity Among Inactive Community-Dwelling People Aged 65 years or More: Protocol for a Randomized Controlled Trial
Source: JMIR Res Protoc. 2025 Jun 20;14:e68796. doi: 10.2196/68796 (PMC12228010; doi:10.2196/68796)
Supplement: Multimedia Appendix 5 [file resprot_v14i1e68796_app5.pdf]

GRB ASSESSMENT REPORT – GRANT APPLICATION

**Application No.:**	20210531
**Project Title:**	A randomized controlled trial evaluating a Trans-Theoretical-Model-based online intervention delivered by fully-automated Chatbot in increasing physical activity among inactive community-dwelling people aged 65 years
**Principal Applicant (PA):**	Dr WANG Zixin
**Rating:**	3. Recommended for support subject to clarifications/ amendments

Comments

1. Clarify the feasibility, generalisability and the potential for the findings of this study to inform clinical practice.

2. Justify the sample size.

3. Clarify the intervention period and follow-up period.

4. Justify the 12-week intervention is needed

5. Human research and survey research ethics approvals are required.

Reviewers’ comments

• Refer and respond to the Reviewers' comments point-by-point.

Action required by PA

• Please refer to the checklist at

https://rfs.healthbureau.gov.hk/images/HMRF/Checklist_for_fundable_projects.pdf for details in preparing your response.

• Respond to the GRB's and Reviewers' comments point-by-point.

• Submit a revised "Proposed Research Project", i.e. Section 13 of Application Form, in pdf files (both "track change" version and clean copy are required).

• Submit all the materials required via eGMS by **9 November 2022**.

• Human research and survey research ethics approvals shall be submitted via eGMS no later than **11 January 2023**.

Grading for applications	Meaning
**4**<br>**Recommended for support**	Nil or very minor issues to address only
**3**<br>**Recommended for support subject to clarifications / amendments**	Minor revision and clarification required for a successful delivery
**2**<br>**Not recommended for support at present**	Major revision required for significant improvement
**1**<br>**Not supported**	Minimal impact on research/flaw in methodology/incomplete application/ out of scope of the fund

REFEREE'S ASSESSMENT FORM

Reference No.: 20210531

Project Title: A randomized controlled trial evaluating a Trans-Theoretical-Model-based online intervention delivered by fully-automated Chatbot in increasing physical activity among inactive community-dwelling people aged 65 years

PART A: REFEREE'S DETAILED REMARKS ON THE INDIVIDUAL SECTIONS OF THE GRANT APPLICATION

1. **Originality and Impact** What is the importance of the proposed research in terms of its originality and potential impact in the area under study? How will the research findings benefit patients and/or the healthcare system? Will the research findings improve patient care, population health, influence clinical practice and/or health services management, or inform health policy in Hong Kong and elsewhere? Have the potential facilitators and barriers to this impact being achieved been identified?

The original aspect of the proposed research is use of artificial intelligence in the form of an automated Chatbot to increase physical activity in inactive community-dwelling older adults. In additional, there are limited studies that apply the stages of change behavourial change theory to health promotion and physical activity interventions, which this study proposes to do. Because of the clearly known importance of physical activity on health in older adults, and the evidence that a substantial proportion of older adults are not meeting current physical activity guidelines, this study has the potential to improve an important behaviour in a population that will benefit from it. In the Introduction section, I would have liked to see more evidence from peer reviewed human studies on the effectiveness of artificial intelligence technologies such as Chatbot on changing human behaviour – this is suggested but it appears that this evidence is still limited and in the early stages of research.

2. **Research Questions, Aims and Hypotheses** How specific, clearly expressed and realistic are the research questions, aims and hypotheses?

The research aim is clearly written and is consistent for an RCT design. The 2 experimental arms, primary outcome, follow up duration, and sample description defined are clearly written within the primary research aim. Similarly the primary hypothesis was clearly written.

3. **Subjects and Study Methodology** (i) Is the proposed design and methodology appropriate for the study? (ii) Are sample sizes clear, justified, adequate and realistic? (iii) Are any preliminary data available? (iv) How feasible is the proposed timeframe? (v) Please also provide comments on the following (where applicable):

   • For proposals submitted under Advanced Medical Research (refer to Area of Project on page 1 of application), is this a clinical study which applies advanced technologies including but not limited to biotechnology in medicine, use of drugs and treatments, clinical trials, virtual health such as telemedicine, etc., to facilitate the translation of knowledge generated from health and health services or infectious diseases studies into clinical practice and to inform health policy?

• For proposals addressing thematic priorities under Implementation Science (refer to Section 5b of the application), are the appropriate framework(s) / model(s) with the pre-set criteria proposed to evaluate/assess the barriers and facilitators of implementation outcomes clearly stated?

• For Seed Grant proposals (i.e. grant ceiling is HK$500,000), is the prospect that a successful outcome will enable scale-up to a larger project/trial and/or enhance the efficacy/effectiveness of existing practice clearly stated and feasible?

Yes, the randomized controlled trial design is appropriate. The RCT is not blinded, and I believe this is fine and appropriate for such a behaviour study where the 2 experimental arms are different and it is not possible to maintain blinding of both participants and study personnel during their follow up communications. Methodologic rigour to the RCT is added with random allocation being concealed from the research personnel because the Chatbot administers the random allocation process.

The sample size calculation was well detailed and justified and appears to be adequate with respect to the primary study outcome at 6 months. The researchers expected a 30% loss to follow up; this sounds reasonable but it would have been helpful for the researchers to expand on their reasons for this prediction.

No preliminary data for this study was provided.

The timeline is reasonable; however, I would say it is rather tight. The researchers project that they will hit their recruitment target of 278 participants in 6 months, citing another of their studies in which they were able to recruit 396 participants in 4 months. However, I would have liked to read more detail about this previous study -how did the eligibility criteria differ to that of the present study and when (what year(s)) was this previous study done – with COVID sometimes people are less available due to illness, or less open altogether, in engaging in additional activities.

4. **Outcomes and Data Analysis** (i) Are the primary and secondary outcomes clearly defined? (ii) Have potential problems been anticipated and addressed? (iii) Is the statistical/analytical design appropriate and clearly explained?

Yes the primary and secondary outcomes are clearly defined. However, I would have liked to see more rationale provided about the decision for a 6 month follow up. Why not a longer follow up such as 12 months, despite the costs/feasibility issues of extending a study past 2 years. From a clinically meaningful perspective, a longer than 6 month follow up would add value to the literature because there is a dearth of information on longer term impact of behavioural interventions. In addition, if participants will be wearing the accelerometer for 7 days at T0, and just after T1 and just after T2, are there concerns that participant behaviour will be altered (eg more favorable) while wearing the accelerometer compared with when not required to wear it. The statistical and analytical design is appropriate and well detailed. Intention to treat analysis will be used, which is a strength.

5. **Research Capability** Comment on (i) the research team's expertise and track record (incl. principal investigator / project team members / collaborators) and (ii) the existing facilities of the Institution where the research will be conducted.

The research team has a strong research track record and each member on the team has expertise that will complement that content areas of the study. The researchers have previous experience in Chatbot development and study. The existing facilities are equally suitable and strong. Overall, I have no concerns about the research capability or facilities of the team.

6. **Budget** Is the request for research personnel, consumables, equipment and overall budget justified and reasonable? [For reference, 1 USD is equivalent to approximately 7.8 HKD]

The budget is sufficiently detailed. A large proportion of the budgetary items are staff related, which is reasonable due to the large sample size and the field work as well as participant contact work involved. Overall, I think the budget is reasonable with respect to the nature of the study.

7. **Ethical and Safety Considerations** Is the proposed research ethically sound? Outline any safety or ethical issues that from the proposed research and comment on whether these have been adequately addressed in the proposal. Has ethical approval been sought?

The researchers indicate that human research ethics approval and survey research ethics approval will be sought. The study itself is relatively low risk to participants, however, I would have liked to see more detail about the estimated potential for harm or injury in engaging in physical activity (eg potential for falls, etc.) for those who were inactive at baseline, and how the researchers plan to mitigate and handle these potential issues.

8. **Overall Comments and Conclusion** It is always helpful for applicants to receive constructive feedback from reviewers. What are the specific strengths and weaknesses of this proposal? Please include a brief overall appraisal of the proposal focusing on any areas for improvement and the basis for your comments, e.g. awareness of other work in the field.
Strengths:

Strengths – overall, the study proposes to use a strong research design, a sufficient sample size, and a relatively accessible/feasible intervention to improve an important health behaviour (physical activity) in a population in which physical activity is largely inadequate and for which there is great potential benefits (inactive community dwelling older adults). The research team has the experience and track record to successfully complete this type of project.

Weaknesses:

Weaknesses – The study could better address the rationale for selecting a 6 month follow up time (rather than a longer follow up period such as 9 or 12 months). In addition, the study rationale would be strengthened by providing more details from human research evidence to support the efficacy of artificial intelligence technology on changing human behavior.

REFEREE'S ASSESSMENT FORM

Reference No.: 20210531

Project Title: A randomized controlled trial evaluating a Trans-Theoretical-Model-based online intervention delivered by fully-automated Chatbot in increasing physical activity among inactive community-dwelling people aged 65 years

PART A: REFEREE'S DETAILED REMARKS ON THE INDIVIDUAL SECTIONS OF THE GRANT APPLICATION

1. **Originality and Impact** What is the importance of the proposed research in terms of its originality and potential impact in the area under study? How will the research findings benefit patients and/or the healthcare system? Will the research findings improve patient care, population health, influence clinical practice and/or health services management, or inform health policy in Hong Kong and elsewhere? Have the potential facilitators and barriers to this impact being achieved been identified?

This project aims to investigate a novel approach to increasing physical activity (PA) in the elderly population in Hong Kong. The lack of PA in the public at large is a perennial problem and no study has so far been able to successfully resolve the issue. The authors give a nice background of the planned research. They point to an even higher lack of PA in the population of elderly people and higher importance of this lack given the known clinical sequelae of across-the-board increased morbidity and mortality, particularly in the dependence-induced disease domain. The authors cite pros and cons concerning the undertaking of PA by the elderly and the hitherto rather inconclusive and spare randomized clinical trials (RCT) of a variety of methods, including various e-health or e-platforms tried to stimulate the eagerness to perform PA in the elderly. The issue is of paramount health importance for disease prevention, health care for the senior people’s quality of life, health services and costs, clinical management, and socioeconomic aspects, let alone mortality. The issue is potentiated by the ever-increasing size of the elderly group worldwide, also a feature of the Hong Kong community.

Therefore, every effort to investigate how to entice the elderly to PA is of value. The potential positive influence of the study is very big. The proposed trial is justified and welcome, although it is not quite original in terms of the issue to be resolved or the uniqueness of the proposed Trans-Theoretical Model (TTM) intervention. TTM has been increasingly used in health promotion campaigns, particularly to promote various vaccinations. This is a relatively new model which explains the paucity of studies so far. Anyhow, the authors fit well into the current trends of this kind of motivational behavioral enhancement. What is more innovative is the use of Chatbot software based on artificial intelligence that provides automated messages after considering and analyzing responders’ preferences and behaviors; a kind of intelligent human-machine interaction.

This method has also been increasingly used in health prevention of late, although not yet for the enhancement of PA in the elderly.

2. **Research Questions, Aims and Hypotheses** How specific, clearly expressed and realistic are the research questions, aims and hypotheses?

The research aims are clearly stated. The core of the TTM method is going through the stages of change (SOC) phases. The authors proposed the hypothesis that going through a Chatbot-

delivered stage-tailored online intervention would be superior to the non-stage Chatbot-delivered intervention for meeting the WHO recommended level of PA in the inactive elderly population in Hong Kong. Simply put, the people in the intervention group will get personal-tailored feedback-related automated messages as opposed to standard non-tailored messages in the control group and more people in the former group meet the recommended PA than in the latter group. That is all clear, nice, and feasible. What is less clear is the duration of the intervention. The messaging will be held for 3 months, and a 6-month follow-up is considered. I am unclear whether the 3 months of the intervention are included in the 6 months, or the follow-up begins after the end of the 3-month messaging.

3. **Subjects and Study Methodology** (i) Is the proposed design and methodology appropriate for the study? (ii) Are sample sizes clear, justified, adequate and realistic? (iii) Are any preliminary data available? (iv) How feasible is the proposed timeframe? (v) Please also provide comments on the following (where applicable):

• For proposals submitted under Advanced Medical Research (refer to Area of Project on page 1 of application), is this a clinical study which applies advanced technologies including but not limited to biotechnology in medicine, use of drugs and treatments, clinical trials, virtual health such as telemedicine, etc., to facilitate the translation of knowledge generated from health and health services or infectious diseases studies into clinical practice and to inform health policy?

• For proposals addressing thematic priorities under Implementation Science (refer to Section 5b of the application), are the appropriate framework(s) / model(s) with the pre-set criteria proposed to evaluate/assess the barriers and facilitators of implementation outcomes clearly stated?

• For Seed Grant proposals (i.e. grant ceiling is HK$500,000), is the prospect that a successful outcome will enable scale-up to a larger project/trial and/or enhance the efficacy/effectiveness of existing practice clearly stated and feasible?

The study design is innovative. The TTM along with its consecutive SOC stages is the most current method increasingly used for health promotion purposes. The authors add to that the Chatbox artificial intelligence. These are the methods that enhance the effectiveness of existing health promotion practices. The application of the method is feasible in this project. The methodology has already been in use, also in other sister study designs such as antismoking investigations or vaccination promotion areas.
The authors allude to their other investigations using the Chatbox methodology in the anti-smoking, influenza vaccination-promoting, and HIF self-testing investigations. Such investigations may be considered preliminary for the current use of the method for PA promotion among the elderly The authors are well versed in using the method.
Inclusion and exclusion criteria are presented in detail, including the assessment of basal physical activity, cognitive status, and the willingness and agility in using electronic devices for picking up messages. The size of the group considers a 30% dropout rate. After consideration for the statistical power of results and effect size, the authors arrive at the size of the groups of 97 persons each, which seems OK. The timeline of the project covers two years. The distribution of sequential tasks as the study goes does not raise any reservations.

4. **Outcomes and Data Analysis** (i) Are the primary and secondary outcomes clearly defined? (ii) Have potential problems been anticipated and addressed? (iii) Is the statistical/analytical design appropriate and clearly explained?

The primary outcome will be the level of physical activity (PA) before/after the intervention based on the trans-theoretical model delivered by a fully automated Chatbot messaging platform in the intervention versus control groups. PA will be objectively verified by accelerometers placed on wrists. And here come my reservations. Accelerometers have disadvantages in that they cannot record angular motions or light activity as they usually have an inbuilt limit of the activity to be detected. So, the technical details of the accelerometers to be used should be provided. Then, the placement on the wrist and why not on the hip which is usually a more sensitive recording.
I also take issue with having just one method of recording PA, an accelerometer, particularly since it is not perfectly reliable. Why not add a questionnaire assessing PA, along with, say, surveys assessing cognitive status, and motivational aspects, both important and liable to improve after your intervention. That could strengthen the research results and fill a gap of missing any secondary outcome.
The statistical elaboration including the calculation of group size, intention-to-treat, and between-group comparison is up-todate and raises no reservations.

5. **Research Capability** Comment on (i) the research team's expertise and track record (incl. principal investigator / project team members / collaborators) and (ii) the existing facilities of the Institution where the research will be conducted.

This research project will be conducted at the JC School of Public Health and Primary Care of the Chinese University of Hong Kong, a renowned institution, endowed with this kind of neuropsychological and epidemiological research methodology. The school's staff consists of a variety of public-community health as well as clinical specialists, including biostatistics which is an essential part of this project. Likewise, the remaining 3 team members engaged in the project have an excellent track record of publications as well as previous or concurrent grants. Prof. Zixin Wang, the team's leader has expertise in clinical trials and eHealth interventions, also those using the Chatbox methodology, referring to other health promotion issues

6. **Budget** Is the request for research personnel, consumables, equipment and overall budget justified and reasonable?
[For reference, 1 USD is equivalent to approximately 7.8 HKD]

The budget sheet details all the different expenses and seems reasonable given the 2-year long research requiring a great number of different activities, statistical elaboration, post-research dissemination of results, etc.

7. **Ethical and Safety Considerations** Is the proposed research ethically sound? Outline any safety or ethical issues that from the proposed research and comment on whether these have been adequately addressed in the proposal. Has ethical approval been sought?

On the ethical side, the project is noninvasive, completely safe, and does not require any special precautions. The authors address the need for obtaining consent from the prospective participants and for research involving human survey-like research.

8. **Overall Comments and Conclusion** It is always helpful for applicants to receive constructive feedback from reviewers. What are the specific strengths and weaknesses of this proposal? Please

include a brief overall appraisal of the proposal focusing on any areas for improvement and the basis for your comments, e.g. awareness of other work in the field.

Strengths:

Use of artificial intelligence-enhanced patient-tailored e-communication platform to perform a clinical trial and thus a chance to collect evidence-based results.<br>Importance for community health services.<br>Reducing morbidity and related mortality of the elderly.<br>Positive socio-economic outcome.<br>Extending lifespan and particularly health span of the elderly.<br>Health promotion.<br>Fits well into anti-aging physical rehabilitation.

Weaknesses:

The use of an accelerometer only to assess physical activity (PA), a device having some shortcomings discussed in ‘Outcomes’.<br>Lack of a confirmatory psychometric instrument to assess PA and cognitive and motivational statuses of participants after the intervention.

REFEREE'S ASSESSMENT FORM

R eference No.: 20210531

Project Title: A randomized controlled trial evaluating a Trans-Theoretical-Model-based online intervention delivered by fully-automated Chatbot in increasing physical activity among inactive community-dwelling people aged 65 years

PART A: REFEREE'S DETAILED REMARKS ON THE INDIVIDUAL SECTIONS OF THE GRANT APPLICATION

1. **Originality and Impact** What is the importance of the proposed research in terms of its originality and potential impact in the area under study? How will the research findings benefit patients and/or the healthcare system? Will the research findings improve patient care, population health, influence clinical practice and/or health services management, or inform health policy in Hong Kong and elsewhere? Have the potential facilitators and barriers to this impact being achieved been identified?

   This proposed RCT aims to address the research gap - no online physical activity (PA) RCT intervention conducted in older Chinese adults, even though 18 relevant RCT studies have been conducted worldwide. Furthermore, limited relevant studies were guided by the theory, such as the Trans-Theoretical Model (TTM). The research team used literature and the team's previous work on older adults to make a good argument for the need of the proposed RCT study.

2. **Research Questions, Aims and Hypotheses** How specific, clearly expressed and realistic are the research questions, aims and hypotheses?

   Appropriate.

3. **Subjects and Study Methodology** (i) Is the proposed design and methodology appropriate for the study? (ii) Are sample sizes clear, justified, adequate and realistic? (iii) Are any preliminary data available? (iv) How feasible is the proposed timeframe? (v) Please also provide comments on the following (where applicable):

   • For proposals submitted under Advanced Medical Research (refer to Area of Project on page 1 of application), is this a clinical study which applies advanced technologies including but not limited to biotechnology in medicine, use of drugs and treatments, clinical trials, virtual health such as telemedicine, etc., to facilitate the translation of knowledge generated from health and health services or infectious diseases studies into clinical practice and to inform health policy?

   • For proposals addressing thematic priorities under Implementation Science (refer to Section 5b of the application), are the appropriate framework(s) / model(s) with the pre-set criteria proposed to evaluate/assess the barriers and facilitators of implementation outcomes clearly stated?

   • For Seed Grant proposals (i.e. grant ceiling is HK$500,000), is the prospect that a successful outcome will enable scale-up to a larger project/trial and/or enhance the efficacy/effectiveness of existing practice clearly stated and feasible?

The proposed 2-arm RCT is appropriate.
The followings are the suggestions and comments:
1. As mentioned in the proposal, the research team "will pilot the Chatbot among another 30 older adults to improve its accuracy of human-Chatbot interactions". Also, a pilot study will be conducted with 6 eligible participants to test the logistics of the intervention. Although the research team has the experience to develop Chatbot interventions for smoking cessation, seasonal influenza, pneumococcal vaccination, and home-based HIV self-testing, the content area of the current proposed study is different from those previous works. It is not clear if the online videos have been developed or validated. This is the main weakness of the proposal.
2. It is not clear why the 12-week duration is selected since not much discussion has been provided in the proposal.
3. It is not clear if two follow-ups will be conducted at Month 3 and Month 6 (as shown in Figure 2 Flow Chart of the RCT), but the research questions only mentioned Month 6.
4. It seems all the briefings will be conducted online. It is not clear how to distribute the accelerometer to the participants

4. **Outcomes and Data Analysis** (i) Are the primary and secondary outcomes clearly defined? (ii) Have potential problems been anticipated and addressed? (iii) Is the statistical/analytical design appropriate and clearly explained?

Appropriate.

5. **Research Capability** Comment on (i) the research team's expertise and track record (incl. principal investigator / project team members / collaborators) and (ii) the existing facilities of the Institution where the research will be conducted.

Appropriate with experience in developing AI-Chatbot.

6. **Budget** Is the request for research personnel, consumables, equipment and overall budget justified and reasonable?
[For reference, 1 USD is equivalent to approximately 7.8 HKD]

Appropriate.

7. **Ethical and Safety Considerations** Is the proposed research ethically sound? Outline any safety or ethical issues that from the proposed research and comment on whether these have been adequately addressed in the proposal. Has ethical approval been sought?

Need to indicate how to provide support to the participants if they encounter health problems during exercise.

8. **Overall Comments and Conclusion** It is always helpful for applicants to receive constructive feedback from reviewers. What are the specific strengths and weaknesses of this proposal? Please include a brief overall appraisal of the proposal focusing on any areas for improvement and the basis for your comments, e.g. awareness of other work in the field.
Strengths:

The research team has experience in conducting AI-Chatbot research.<br>The proposed RCT addresses the research gap.

Weaknesses:

See above.

REFEREE'S ASSESSMENT FORM

R eference No.: 20210531

Project Title: A randomized controlled trial evaluating a Trans-Theoretical-Model-based online intervention delivered by fully-automated Chatbot in increasing physical activity among inactive community-dwelling people aged 65 years

PART A: REFEREE'S DETAILED REMARKS ON THE INDIVIDUAL SECTIONS OF THE GRANT APPLICATION

1. **Originality and Impact** What is the importance of the proposed research in terms of its originality and potential impact in the area under study? How will the research findings benefit patients and/or the healthcare system? Will the research findings improve patient care, population health, influence clinical practice and/or health services management, or inform health policy in Hong Kong and elsewhere? Have the potential facilitators and barriers to this impact being achieved been identified?

The proposal is for an RCT evaluating a Trans-Theoretical-Model-based intervention delivered by Chatbot to increase physical activity (measured with accelerometers). The aim is to compare a stage-tailored intervention to a non-stage-tailored intervention.

2. **Research Questions, Aims and Hypotheses** How specific, clearly expressed and realistic are the research questions, aims and hypotheses?

The hypothesis and research questions are clear.

3. **Subjects and Study Methodology** (i) Is the proposed design and methodology appropriate for the study? (ii) Are sample sizes clear, justified, adequate and realistic? (iii) Are any preliminary data available? (iv) How feasible is the proposed timeframe? (v) Please also provide comments on the following (where applicable):

   • For proposals submitted under Advanced Medical Research (refer to Area of Project on page 1 of application), is this a clinical study which applies advanced technologies including but not limited to biotechnology in medicine, use of drugs and treatments, clinical trials, virtual health such as telemedicine, etc., to facilitate the translation of knowledge generated from health and health services or infectious diseases studies into clinical practice and to inform health policy?

   • For proposals addressing thematic priorities under Implementation Science (refer to Section 5b of the application), are the appropriate framework(s) / model(s) with the pre-set criteria proposed to evaluate/assess the barriers and facilitators of implementation outcomes clearly stated?

   • For Seed Grant proposals (i.e. grant ceiling is HK$500,000), is the prospect that a successful outcome will enable scale-up to a larger project/trial and/or enhance the efficacy/effectiveness of existing practice clearly stated and feasible?

The proposed study design is generally appropriate. However, the outcome is defined as dichotomous (meeting WHO recommended levels of PA); more study power would be gained

by also measuring the outcome as a continuous variable. It is not appropriate participants who do not wear the accelerometer are excluded – surely this is the group that probably also has the lowest levels of physical activity, and which is not following the intervention. An intention-to-treat analysis would require that they are retained in the analysis, and their PA is still measured at the end of the intervention.

4. **Outcomes and Data Analysis** (i) Are the primary and secondary outcomes clearly defined? (ii) Have potential problems been anticipated and addressed? (iii) Is the statistical/analytical design appropriate and clearly explained?

The application states that an intention-to-treat analysis will be conducted – so all participants should be retained, whether or not they follow the intervention and wear the accelerometer.

5. **Research Capability** Comment on (i) the research team's expertise and track record (incl. principal investigator / project team members / collaborators) and (ii) the existing facilities of the Institution where the research will be conducted.

The team has the necessary expertise and experience.

6. **Budget** Is the request for research personnel, consumables, equipment and overall budget justified and reasonable?
[For reference, 1 USD is equivalent to approximately 7.8 HKD]

No concerns.

7. **Ethical and Safety Considerations** Is the proposed research ethically sound? Outline any safety or ethical issues that from the proposed research and comment on whether these have been adequately addressed in the proposal. Has ethical approval been sought?

No concerns.

8. **Overall Comments and Conclusion** It is always helpful for applicants to receive constructive feedback from reviewers. What are the specific strengths and weaknesses of this proposal? Please include a brief overall appraisal of the proposal focusing on any areas for improvement and the basis for your comments, e.g. awareness of other work in the field.
Strengths:

This is an interesting topic, and potentially important, and the study design, and the proposed methods of data analysis, are generally appropriate.

Weaknesses:

It is not appropriate to exclude those who do not wear the accelerometers.
